# Implementing the PHMRC shortened questionnaire: Survey duration of open and closed questions in three sites

**DOI:** 10.1371/journal.pone.0178085

**Published:** 2017-06-01

**Authors:** Abraham D. Flaxman, Andrea Stewart, Jonathan C. Joseph, Nurul Alam, Saidul Alam, Hafizur Chowdhury, Saman Gamage, Hebe Gouda, Rohina Joshi, Marilla Lucero, Meghan D. Mooney, Devarsetty Praveen, Rasika Rampatige, Hazel Remolador, Diozele Sanvictores, Peter T. Serina, Peter Kim Streatfield, Veronica Tallo, Nandalal Wijesekera, Christopher J. L. Murray, Bernardo Hernandez, Alan D. Lopez, Ian Douglas Riley

**Affiliations:** 1 Institute for Health Metrics and Evaluation, University of Washington, Seattle, Washington, United States of America; 2 International Centre for Diarrhoeal Disease Research, Dhaka, Bangladesh; 3 School of Population and Global Health, University of Melbourne, Parkville, VIC, Australia; 4 WHO Collaborating Centre for Public Health Workforce Development, National Institute of Health Sciences, Kalutara, Sri Lanka; 5 Papua New Guinea Institute of Medical Research, Goroka, Papua New Guinea; 6 School of Public Health, University of Queensland, QLD, Australia; 7 Queensland Centre for Mental Health Research, QLD, Australia; 8 The George Institute for Global Health, Sydney, Australia; 9 Research Institute for Tropical Medicine, Muntinlupa City, Philippines; 10 George Institute of Global Health India, Hyderabad, India; BC Children's Hospital, CANADA

## Abstract

**Background:**

More countries are using verbal autopsy as a part of routine mortality surveillance. The length of time required to complete a verbal autopsy interview is a key logistical consideration for planning large-scale surveillance.

**Methods:**

We use the PHMRC shortened questionnaire to conduct verbal autopsy interviews at three sites and collect data on the length of time required to complete the interview. This instrument uses a novel checklist of keywords to capture relevant information from the open response. The open response section is timed separately from the section consisting of closed questions.

**Results:**

We found the median time to complete the entire interview was approximately 25 minutes and did not vary substantially by age-specific module. The median time for the open response section was approximately 4 minutes and 60% of interviewees mentioned at least one keyword within the open response section.

**Conclusions:**

The length of time required to complete the interview was short enough for large-scale routine use. The open-response section did not add a substantial amount of time and provided useful information which can be used to increase the accuracy of the predictions of the cause of death. The novel checklist approach further reduces the burden of transcribing and translating a large amount of free text. This makes the PHMRC instrument ideal for national mortality surveillance.

## Introduction

Cause of death information is the cornerstone of a national health information system and is crucial for evaluating the impact of health policies and informing priority setting in the health sector. [1] However, cause of death data are either not available, or of very poor quality, in many parts of the world. [2] Ideally cause of death information should be based on accurate medical certification of each death in the population by a qualified medical practitioner and recorded promptly and correctly in national vital registration systems. [3] Some countries have made progress in improving their cause of death data systems, but the majority have not. [4]

Given the widespread lack of medical doctors to certify deaths in many countries, and the likelihood that the availability of trained medical certifiers will not increase very rapidly, there is increasing interest in the use of verbal autopsy (VA) methods to provide governments with actionable cause of death data in many countries [5]. The World Health Organization has called for expanded use of VA to provide population level estimates of cause of death. [6] Some countries, such as India[7], Bangladesh [8], Brazil [9], China [10], and Tanzania [11], have already incorporated VA into routine health surveillance and national vital registration systems.

The time and cost of collecting VA data is an important consideration, especially in low and middle income countries where the data are most needed and resources are scarce. Three ways to minimize the time and cost of collecting and coding VAs are through automated algorithms for coding the cause of death, using electronic devices for data collection, and shortened interviews. Development of automated computer algorithms for diagnosing the cause of death from verbal autopsy interviews (VAI) is now sufficiently advanced that it can replace physician coding of VA and achieve more reliable and accurate results, at only a fraction of the cost and with minimal time delays. [12] Direct data capture using electronic devices eliminates the need to transcribe paper interviews which allows the data to be analyzed shortly after it is collected and reduces transcription errors. [13] Automated diagnostic methods applied to VAIs collected on a tablet or smartphone greatly reduces the time and cost of analyzing data. However, for routine application in national vital registration systems, it is also important to reduce the time and costs associated with collecting interviews. The length and structure of a VA questionnaire greatly determines the duration of data collection and is an important consideration in scaling up verbal autopsy in national vital registration systems. Open responses narratives often have to be translated and transcribed, also increasing the time for data entry. Previous research has found that an item-reduced VA questionnaire which includes an open ended narrative section shows only a very small drop in overall accuracy, compared with the much longer VA questionnaires, when analyzed by automated computer coding. [14]

In this study we measured the time needed to complete verbal autopsy interviews using the Population Health Metrics Research Consortium (PHMRC) Shortened Questionnaire, based on application of the methods in three countries. We also compared the time required for completing the close-ended (structured) questions in the VAI to the time needed to record the open narrative when using a novel “narrative checklist” approach. The length of time required to obtain useful VAIs is critically important information for guiding strategies to scale up VA methods to meet the needs of national vital registration systems and for reducing interview fatigue for the relatives of the deceased.

## Materials and methods

### Instrument

The VA questionnaire used in this study is a shortened form of the questionnaire developed for the Population Health Metrics Research Consortium Gold Standard Verbal Autopsy Validation study (PHMRC study). [15] The PHMRC VAI consists of two sections. The first is a number of close-ended questions about the decedent which covers symptoms of the terminal illness, preexisting chronic illnesses, and health service interactions. In the second part, known as the open narrative, the respondent is asked to describe in their own words the events leading up to the death of the decedent. Each VAI consists of a general module and an age-specific module depending on whether the decedent was an adult, child or neonate; the age-specific modules contain skip logic to include certain sections only when relevant (for example, for injury deaths or deaths in women of reproductive age).

The original PHMRC VA questionnaire was shortened to include only the most relevant questions, based on empirical evidence. The process for creating a shortened instrument based on the full length questionnaire is described in detail elsewhere. [14] Briefly, questions from the PHMRC Full Questionnaire were ranked in terms of their importance for distinguishing different causes of death using the Tariff 2.0 automated method of analysis. [16] The least important questions were dropped one at a time and the predictive performance of algorithm at the individual and population levels were assessed. Questions were dropped until an optimum size was reached as determined by a first derivative analysis of the rate of decline in predictive accuracy.

For the shortened questionnaire, the set of questions about injuries was moved to earlier in the survey. Injury related deaths are often very clearly known and well reported. When the respondent reports on an injury that led to death, additional questions about signs and symptoms are largely irrelevant. To streamline the interview process, injury questions were asked before signs and symptoms and, if the death was the result of an injury, the instrument employs a skip pattern where the sections on signs and symptoms are avoided since they are irrelevant to injury deaths.

The open narrative section of the VAI provides important additional information. Sometimes respondents mention key words related to diseases, even if they failed to endorse close-ended questions related to the same diseases or symptoms. However, previous experiences showed that entering open-ended responses on tablet devices was challenging, especially in areas where there are numerous languages and/or different alphabet scripts. This resulted in the interviewer recording the open response on paper and then transcribing it onto the tablet later, adding additional time and burden on to the interview process. Automated computer coding algorithms use a text mining approach to analyze the open narrative section.[16] We used a checkbox approach for capturing information from the open response section developed previously, [ref] to eliminate the need to transcribe and translate responses while still providing the relevant information for computer algorithms which code the cause of death. The checkbox approach works by asking interviewer to listen for certain keywords and check a box for each word mentioned. A list of important keywords were identified (included for convenience in Table 1; a detailed description of the keyword identification process has been published elsewhere [14]). Interviewers were asked to listen for the keywords during the open response part of the interview and check a box if the key word was mentioned. The list of words chosen was based on tariffs and their importance for distinguishing causes of death which are important for public health. The number of items was limited so that all the words could fit on one screen of the data collection device and interviewers could easily remember the entire list. This included eleven words for the adult module, ten words for the child module and six words for the neonate module. Table 1 shows the key words selected for each module.

**Table 1:** Keywords identified and used in the open response narrative section.

Adult	Child	Neonate
Chronic Kidney Disease	Abdomen	Asphyxia (lack of oxygen)
Dialysis	Cancer	Incubator
Fever	Dehydration	Lung Problems
Heart Attack (AMI)	Dengue Fever	Pneumonia
Heart Problems	Diarrhea	Preterm Delivery
Jaundice	Fever	Respiratory Distress
Liver Failure	Heart Problems	
Malaria	Jaundice (yellow skin or eyes)	
Pneumonia	Pneumonia	
Renal (kidney) failure	Rash	
Suicide		

### Data collection

Data for this study was all gathered using the shortened version of the questionnaire with a checkbox list of keywords for the open response implemented with the Open Data Kit Collect software and was administered on Samsung Galaxy Tab 2 tablet devices. The survey was fielded at three sites, Matlab, Bangladesh; Bohol, Philippines; and Kalutara, Sri Lanka between May 2013 and July 2013. In Matlab, Bangladesh, the data was collected through the Matlab Health and Demographic Surveillance System (HDSS), Centre for Population, Urbanization and Climate Change (CPUCC), icddr,b, Bangladesh, which is a routine demographic surveillance site. In the Philippines, the data was collected through the Research Institute for Tropical Medicine at the Department of Health in Bohol, an island province of 1.2 million. In Kalutara, Sri Lanka, the data was collected through the National Institute of Health Sciences. Interviewers received a standard training from site supervisors which included thoroughly reviewing the instrument and all technical words from the questions in all the local languages, demonstrations and practice using the instrument on the tablets, and practice conducting full interviews. The methods of this study were approved by the Medical Research Ethics Committee of the University of Queensland, Australia; the National Institute of Health Sciences (NHIS), Sri Lanka; the Institutional Review Board of the Research Institute of Tropical Medicine, Philippines; and by the Ethical Review Committee of the International Centre for Diarrhoeal Disease Research, Bangladesh. All data were collected with informed consent from participants. Written documentation of informed consent was collected in Matlab, Bangladesh and Bohol, Philippines. In Kalutara, Sri Lanka, informed consent was provided verbally and documented on the tablet to reduce operational overhead.

The PHMRC Shortened Questionnaire used for the VAI has been published in English previously. [14] The translations used in Matlab and Bohol are included as supporting information S1 File. In Kalutara, interviewers used the English translation due to a technical limitation that prevented tablets from displaying Sri Lankan script (since remedied).

Time of interview was measured automatically using the tablet. The built-in clock recorded the timestamp of when interview began and when the interviewer closed the electronic form on the tablet. The time needed for the closed and open-ended sections was record separately. The standard protocol is to ask the closed-ended questions first and then ask the open-ended response. However, in Sri Lanka, 72% of the usable observations (63/87) conducted the open response section before the closed-ended questions. Also at this site, unlike the others, prior appointments were scheduled with family members for most interviews.

### Analysis

**Fig 1:**
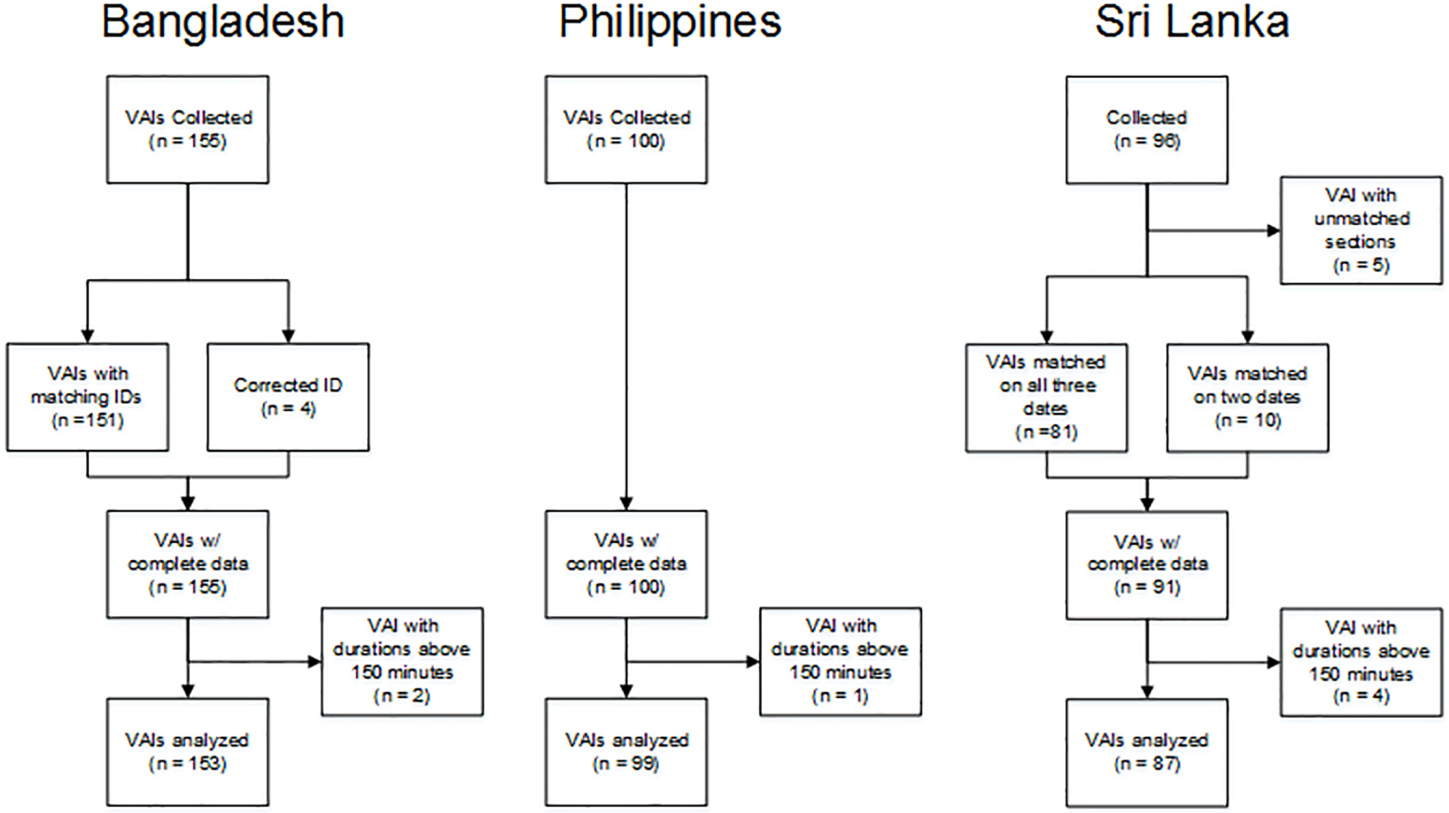
Diagram showing the number of observations used in the analysis by site. In the first step data from the closed and open sections are linked. The data from Sri Lanka did not have a unique identifier and was matched on interview date, birth date and death date.

Records from the closed- and open-ended sections were linked by unique identifiers provided by the sites. Data from Sri Lanka did not include a unique identifier. Therefore records were matched using interview date, birth date and death date. After matching on the full identifier, the remaining records were examined. In most case the identifiers matched on all but one field, such as birth year. It was assumed that this was a transcription error and corresponding records were linked. The remaining observations were dropped. The unique identifier for four observations from the Philippines datasets were corrected by changing one character, usually punctuation, to produce complete matches. Fig 1 shows the loss of records from this process. Only five records from Sri Lanka were dropped due to missing record linkages. The reported interview durations from all sites were examined for outliers. Records were dropped if either section had a duration of greater than 150 minutes. The number of answered and endorsed questions was calculated from the age-specific modules and did not include the questions in the general module. Answers of “Don’t Know” and “Refused to answer” were treated the same as answering “no”. Questions asking about length of time were only considered answered and endorsed if there was a valid time recorded after the screening question.

The median and the interquartile range of the durations were calculated for the closed questions, the open response section, and the total interview comprising the amount of time spent on both sections. These were calculated separately for each module, for each site, and for all observations together. Since the data collection process for Sri Lanka was slightly different, the medians and interquartile ranges of data from the other two sites combined was also calculated. The Sri Lanka data only included adults so this was only relevant for data from the adult module. This instrument skips a large number of questions if injuries are endorsed. Median and interquartile ranges for durations of adults VAI with and without injuries were calculated to show the effect of the skip logic. There were too few injuries reported in the child module to do a similar analysis.

## Results

A total of 351 VAIs were collected from the three sites: 155 from Bangladesh, 100 from the Philippines, and 96 from Sri Lanka. Of these, 290 were adult interviews, 29 were child deaths, and 32 were deaths of neonates. In Bangladesh, two adult VAIs were more than 150 minutes. In the Philippines, one child VAI was reported to have taken more than 150 minutes. In Sri Lanka, four adult VAIs were above the 150 minute cutoff and five adult records did not have matching closed- and open- sections. These observations were dropped giving a total sample size 339 across the three sites.

Across all three sites, and age groups, the median time taken for the total VAI was 23.0 minutes. The median times for the closed- and open-ended sections were 18.4 and 3.5 minutes respectively. The duration of the closed-ended (structured question) response ranged from 4.3 minutes to 77.0 minutes and the open-ended response ranged from 1.0 minute to 25.7 minutes. Table 2 shows the median duration and interquartile range of VAIs by site and module.

**Table 2:** Median and interquartile range of the duration in minutes of the closed- and open-ended sections by site, module and injury endorsement.

	Total		Closed		Open	
	Median	IQR	Median	IQR	Median	IQR
Total (N = 339)	23.0	16.8–31.3	18.4	13.0–25.5	3.5	2.1–5.7
Bangladesh (N = 153)	25.5	18.4–32.3	19.3	14.8–26.9	3.8	2.4–6.4
Philippines (N = 99)	26.0	17.5–31.5	20.0	15.0–25.0	4.0	3.0–6.0
Sri Lanka (N = 87)	17.4	13.3–28.0	14.2	11.1–23.0	2.2	1.5–3.4
Adult (N = 279)	22.3	16.4–31.0	18.0	12.8–25.0	3.1	2.0–5.3
Without Sri Lanka (N = 194)	25.2	17.8–31.3	19.3	14.8–25.0	4.0	2.6–6.8
Adult Injuries (N = 37)	14.4	10.0–18.9	11.5	8.0–14.0	2.6	1.9–4.0
Adult Non-injuries (N = 242)	25.0	17.4–31.6	19.6	14.1–25.9	3.3	2.1–5.7
Child (N = 28)	25.5	17.3–41.2	20.6	15.7–35.5	4.0	2.9–6.3
Neonate (N = 32)	27.8	20.5–34.8	21.0	16.0–30.6	4.0	3.0–6.0

**Fig 2:**
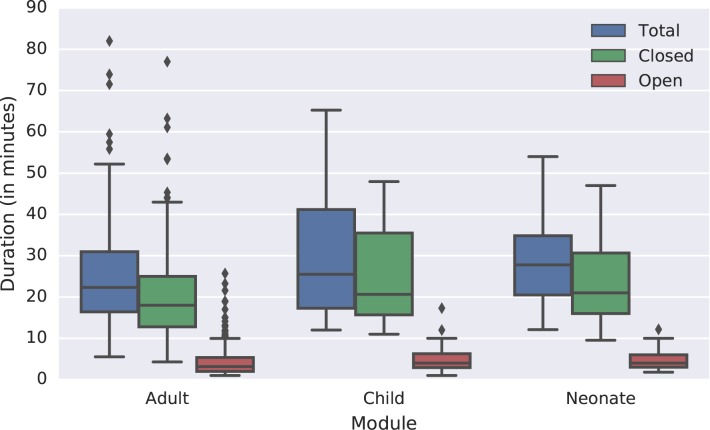
Box plot of length of time needed to complete different verbal autopsy modules.

Sri Lanka had the shortest median duration for both the open and closed section. For the closed section, the median time was 26.6% shorter (5.1 minutes) than Bangladesh and 29.2% shorter (5.8 minutes) than the Philippines. For the open section, the median time was 41.6% shorter (1.6 minutes) than Bangladesh and 45.0% (1.8 minutes) shorter than the Philippines. Fig 2 shows the length of time needed to complete different modules. The adult module had the shortest median duration for both the open and closed section whereas the median durations for the child and neonate modules were approximately the same. For the closed section, the median time for an adult death was 12.8% (2.6 minutes) shorter than the child and 14.3% (3.0 minutes) shorter than neonate modules. For the open section, the median time for adults was 21.3% (0.9 minutes) shorter than both the child and neonate modules.

The median duration of the adult module is likely to have been affected by multiple confounders in the way the data were collected in Sri Lanka, including scheduling interviews ahead of time, conducting the open narrative section first and only including adults. If the durations are analyzed without data from this site the durations are all very similar and the median duration of the adult module is only 9.3% shorter than the neonate module and 1.2% shorter than the child module.

**Fig 3:**
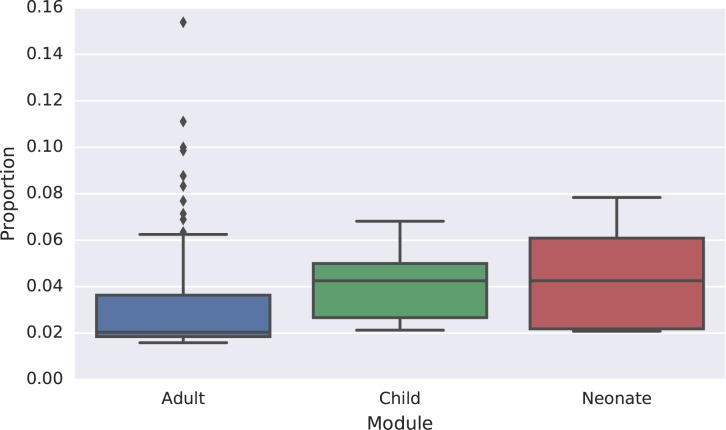
The proportion of the total endorsed symptoms which come from words mentioned in the open narrative section.

Fig 3 shows the proportion of total endorsed symptoms (i.e. items for which the respondent answered ‘yes’) which came from the open response section. The average fraction of endorsed symptoms which were words mentioned in the open response section was 2.0%, ranging between 0 and 16.7%. We found that 60.5% of interviewees mentioned at least one of the key words.

**Fig 4:**
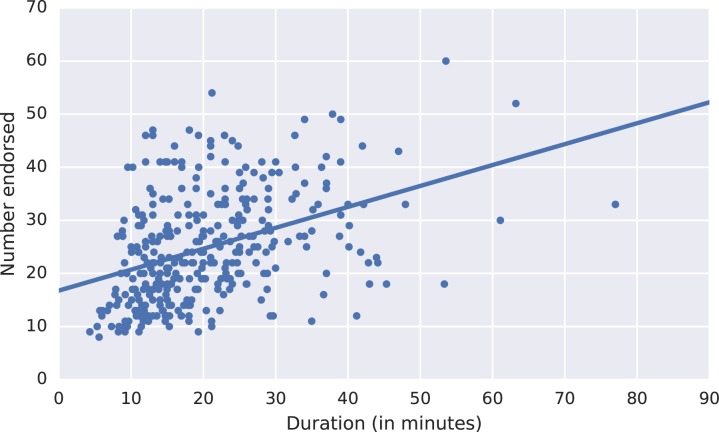
The number of endorsed (i.e. answered “Yes”) closed-ended questions versus the duration of the closed-ended section in all ages.

**Fig 5:**
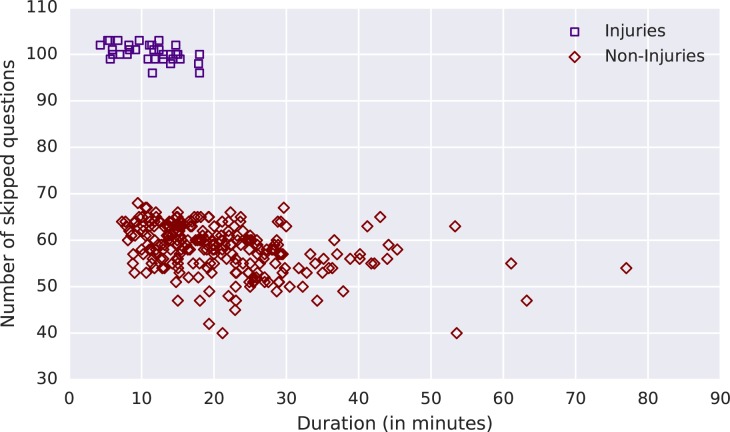
Number of closed-ended question skipped versus the duration of the closed-ended section for adults stratified by whether the death was due to an injury.

The number of questions asked varied due to skip patterns. Fig 4 shows that the length of time needed to complete the closed-ended (structured) question section increased as the number of endorsed questions increases. Questions were considered endorsed if the respondent answered “yes”, but not if the respondent answered “no”, “don’t know” or “refused to answer”. Interviews in which the respondent said the death was the result of an injury were shorter and had smaller variation in duration. Fig 5 shows the number of closed-ended question skipped versus the duration of the closed-ended section for adults. Fewer questions were asked for deaths due to injuries because uninformative signs and symptoms questions were skipped. This results in a shorter interview. The median time for a VAI for adults who died due to injuries was 42.5% (10.6 minutes) shorter than the median time of VAIs for non-injury deaths.

## Discussion

In this study we found that the amount of time required to complete the shortened PHMRC questionnaire including the open narrative section, is approximately 23 minutes. On average, only about 4 minutes was spent on the open narrative section. Two sites, Bangladesh and the Philippines reported very similar interview times, while in Sri Lanka the interview was completed more quickly.

There is some debate about the usefulness of the open narrative section, especially for automatic computer coded verbal autopsies. [17] In particular, the cost of collecting additional information from the open response has to be weighed against the diagnostic value of the additional information provided. Previous research found that over 40% of the informative symptoms were words mentioned during the open response section. [14] Additionally, analyzing the open narrative section led to higher predictive accuracy when using automated computer coding. [14] For these reasons it was included in this questionnaire. Given that the information can be highly informative for determining the cause of death, it is important to capture the information without adding too much additional burden for translating and transcribing text. The checklist approach works as an extremely pragmatic compromise. Despite only having a limited set of key words, over half of those interviewed mention at least one of these words. Also, up to 17% of the usable information came from the open response section. This indicates that the words were not only important for determining the cause of death, but also common enough to be useful when used under real field conditions.

The shorter duration in Sri Lanka may be due to several factors and highlights a number of interesting operational points about conducting VA interviews. The first involves scheduling interviews ahead of time. The last section of the closed-ended questions asks about any health records, and field workers in Sri Lanka found that many interviewees had gathered the medical records of the deceased before the interview. Respondents are asked if they have any health records belonging to the deceased. In Sri Lanka 42% of respondents answered yes to this, compared to only 8% in Bangladesh and none in the Philippines. This may reduce the time needed to complete this section. Also respondents may have re-familiarized themselves with the content of the medical records and events leading to the death of their family member. This may have allowed them to answer questions more rapidly than in families which did not consult medical records. Furthermore, at this site, the study was conducted in an urban setting where the literacy levels and health care experience seemed to be higher.

In Sri Lanka, most of the interviewers began with the open narrative section instead of the closed-ended (structured) section. Some experts suggest that conducting the open response first builds rapport with the interviewee and makes the whole process easier. [18] However, the particular instrument (PHMRC) used in this time study was specifically designed with the open narrative section coming after the closed-ended set of questions about signs and symptoms experienced by the deceased. This matches the order of the WHO instrument. [6] Though the reduced duration is of interest, more work is needed to determine the effect that the reordering the sections might have on predictive accuracy before this can be endorsed.

This study has a number of limitations. First, a relatively small number of verbal autopsy interviews were collected, most of which were adults. It would be important to collect a larger number of VAIs, especially from children and neonates, to provide more accurate and stable estimates of the time required for VAIs. It is also important to collect timing data from more than three sites. Interview processes and cultural familiarity with medical terminology varies widely in different contexts and may influence how much time is required to complete the interview. This study also was not able to quantify the amount of time saved by using the narrative checklist compared to transcribing and translating the open response, or the time saved by using this instrument compared to other instruments.

## Conclusion

This study shows for the first time the length of time required to complete verbal autopsy interviews under real field conditions. This information is critical for governments contemplating scaling up use of VA to provide national level estimates of cause of death for their populations. The amount of time that a VAI typically takes will undoubtedly be an important consideration for governments who wish to incorporate routine VAs on all reported deaths which have not been medically certified. In some countries contemplating such measures the annual number of VA’s might well be as high as 70,000–80,000 and hence the cost effectiveness of the VA methods matters greatly. We believe that the typical time required for the PHMRC VA instrument is sufficiently short to be applied by government vital registration systems. Furthermore, including the open narrative section did not significantly increase the amount of time required but yields useful additional information in at least half the cases. The checklist of key words in the open narrative section included in the PHMRC questionnaire appears to capture the most important diagnostic information, but avoids the burden of transcribing and translating responses. Our findings suggest that the instrument could be readily adopted for widespread use in routine mortality surveillance systems.

## Supporting information

S1 File Translations of PHMRC shortened questionnaire. (PDF)
