# Supplementary material for: Implementing the PHMRC shortened questionnaire: Survey duration of open and closed questions in three sites
Source: PLoS One. 2017 Jun 1;12(6):e0178085. doi: 10.1371/journal.pone.0178085 (PMC5453488; doi:10.1371/journal.pone.0178085)
Supplement: S1 File — (PDF) [file pone.0178085.s001.pdf]

POPULATION HEALTH METRICS RESEARCH CONSORTIUM
NEONATAL AND CHILD VERBAL AUTOPSY MODULE

4 Background Section

4.1 Was the deceased a singleton or multiple birth?
মৃত শিশুটি কি একক না জমজ/একাধিক হিসাবে জন্মেছিল?
এক সাথে দুইজন বা ততোধিক শিশু জন্ম নিলে উহা একাধিক জন্ম হিসেবে গন্য হবে; এমনকি জন্ম নেয়া শিশুদের একজন বা তার বেশী শিশু যদি মৃত জন্মও হয়, যদি একাধিক শিশু জন্ম নেয় তাহলে পরবর্তী প্রশ্ন জিজ্ঞেস করুন।

1. একক
2. একাধিক
8. অনিচ্ছা
9. অজানা

4.2 Was this the first, second or later in the birth order?
এই শিশুটির জন্ম কি প্রথম, দ্বিতীয় বা তৎপরবর্তী ছিল?
যদি মৃত শিশুর মা উপস্থিত থাকেন তাহলে 4.6 নং প্রশ্নে যান আর যদি মা সাক্ষাৎ গ্রহন কালে অনুপস্থিত থাকেন, তাহলে জিজ্ঞেস করুন।

1. প্রথমটি
2. দ্বিতীয়টি
3. তৃতীয়টি বা তার বেশী
8. অনিচ্ছা
9. অজানা

4.3 Is the mother still alive?
মৃত শিশুর মা কি এখনো বেঁচে আছেন?

1. হ্যাঁ
2. না

4.4 Did the mother die during or after the delivery?
মৃত শিশুটির মা প্রসবের সময় না প্রসবের পরে মারা গেছেন?

1. প্রসবের সময়
2. প্রসবের পরে
8. অনিচ্ছা
9. অজানা

Details on death after delivery

4.5 How long after the delivery did the mother die?
প্রসবের কতক্ষণ পরে মা মারা গেছেন?
(২৪ ঘন্টার কম হলে =০০ দিন, মাস হিসাব করতে ১ মাস = ৩০ দিন, এই হিসাবে গননা করুন।)

1. _______ দিন
2. _______ মাস
8. অনিচ্ছা
9. অজানা

4.6 Where was the deceased born?
কোথায় শিশুটির জন্ম হয়েছিল?

1. হাসপাতাল/ICDDR,B
2. অন্যান্য স্বাস্থ্য সেবা প্রতিষ্ঠান
3. হাসপাতাল বা স্বাস্থ্য কেন্দ্রে নেওয়ার পথে
4. বাড়ী
5. অন্যান্য
8. অনিচ্ছা
9. অজানা

4.7 At the time of the delivery what was the size of the deceased? (Read the question and slowly read the first 4 choices. Respondent should hear all four choices and then respond.)
জন্মের সময় মৃত শিশুটি কি রুপ ছিল?
(প্রশ্নটি বলুন এবং প্রথম চারটি উত্তর ধীরে ধীরে বলুন। উত্তরদাতা প্রথমে চারটি উত্তরই জেনে বুঝে উত্তর দিবেন।)

1. খুবই ছোট
2. স্বাভাবিকের চেয়ে ছোট
3. প্রায় স্বাভাবিক
4. স্বাভাবিকের চেয়ে বড়
8. অনিচ্ছা
9. অজানা

Birth Weight details

4.8 What was the weight of the deceased at birth?
জন্মের সময় মৃত শিশুটির ওজন কতটুকু ছিল?

1 ________ গ্রাম
8. অনিচ্ছা
9. অজানা

4.9 Was the child born alive or dead?
শিশুটি কি জীবিত না মৃত হিসাবে জন্মে ছিল?

1. জীবিত
2. মৃত
8. অনিচ্ছা
9. অজানা

4.10 Did the baby ever cry?
শিশুটি কখনো কেঁদেছিল?

1. হ্যাঁ
2. না
8. অনিচ্ছা
9. অজানা

4.11 Did the baby ever move?
শিশুটি কি কখনো নড়েছিল?

1. হ্যাঁ
2. না
8. অনিচ্ছা
9. অজানা

4.12 Did the baby ever breathe?
শিশুটি কি কখনো শ্বাস-প্রশ্বাস ফেলেছিল?

1. হ্যাঁ
2. না
8. অনিচ্ছা
9. অজানা

Decedent never cried, moved, or breathed. (If all three previous responses are `No' then check `Yes' below. Otherwise check `No'.
মৃত শিশুটি যদি একবারও না কাঁদে, না নড়ে, বা শ্বাস না ফেলে তাহলে হাতে নতুবা নাতে লিখুন।

Because the baby never cried, moved, or breathed, this was a stillbirth.
শিশুটি যদি একবারও না কাঁদে, নানড়ে বা শ্বাস না ফেলে তাহলে ইহা মৃত জন্ম

Because the baby either cried, moved, or breathed, this was not a stillbirth.
শিশুটি যদি একবারও কাঁদে, নড়ে বা শ্বাস ফেলে তাহলে ইহা জীবিত জন্ম

4.13 Were there any bruises or signs of injury on the baby's body at birth?
জন্মের সময় বাচ্চাটির গায়ে কোন থেঁতলানো বা আঘাতের চিহ্ন ছিল?

1. হ্যাঁ
2. না
8. অনিচ্ছা
9. অজানা

4.14 Was the baby's body (skin and tissue) pulpy?
বাচ্চাটির চামড়ায় (শরীরে) কোন অংশে ফুলা ছিল?

1. হ্যাঁ
2. না
8. অনিচ্ছা
9. অজানা

4.15 Was any part of the baby physically abnormal at time of delivery?
প্রসবের পর পর বাচ্চাটির শরীরের কোন অংশ অস্বাভাবিক ছিল? (যেমন, কোন অঙ্গ খুব বড় বা ছোট আকৃতির, শরীরে কোন ফুলা বা চাকা

1. হ্যাঁ
2. না
8. অনিচ্ছা
9. অজানা

4.16 What were the abnormalities? (Mark all that apply)
জন্মগত অঙ্গা বিকৃতি গুলি কি কি ছিল?
একাধিক উত্তর হতে পারে।

1. জন্মকালীন অতি ক্ষুদ্রাকৃতির মাথা
2. জন্মকালীন খুব বড়াকৃতির মাথা
3. মাথার পিছনে বা মেরুদন্ডে মাংশপিন্ড
4. অন্যান্য (উলেখ করুন) ____________
8. অনিচ্ছা

What were the abnormalities? (Other abnormality specified)
উত্তর অন্যান্য হলে উলেখ করুন।

Age illness started details

4.17 How old was the baby/child when the fatal illness started?
মারাত্মক অসুখটি যখন শুরু হয় তখন/শিশুটির বয়স কত ছিল?
(২৪ ঘন্টার কম হলে =০০ দিন, মাস হিসাব করতে ১ মাস = ২৮ দিন ধরুন)

1. _______ দিন
(১ মাসের কম হয়)
2. _______ মাস
(যদি ১বছরের কম হয়)
3. _______ বছর
(যদি ১ বছর বা তার বেশী হয়)

4.18 How long did the illness last?
অসুখটি কতক্ষণ স্থায়ী ছিল?
(২৪ ঘন্টার কম হলে =০০ দিন, মাস হিসাব করতে ১ মাস = ২৮ দিন ধরুন)

1. _______ দিন
2. _______ মাস
8. অনিচ্ছা
9. অজানা

4.19 Where did the deceased die?
কোথায় সে মারা গিয়েছিল?

1. হাসপাতাল/ICDDR,B
2. অন্যান্য স্বাস্থ্য সেবা কেন্দ্রে
3. হাসপাতাল বা স্বাস্থ্য কেন্দ্রে নেওয়ার পথে
4. বাড়ীতে
5. অন্যান্য ____________
8. অনিচ্ছা
9. অজানা

Where did the deceased die? (other specified)
অন্যান্য স্থানে হলে নির্দিষ্ট করুন।

4 Maternal history

4.1.1 Was the late part of the pregnancy (defined as the last 3 months) labour or delivery complicated by any of the following problem?
গর্ভাবস্থার শেষের ৩ মাসে প্রসব বা খালাসের সময় নিন্মলিখিত জটিলতার কোনটি হয়েছিল কিনা? (প্রতিটি জটিলতা পড়ে শুনান এবং চিহ্নিত করুন।)
(Read "the mother" if the mother is not the respondant.)

1. আপনার খিঁচুনী ছিল
2.আপনার উচ্চ রক্তচাপ ছিল
3. আপনার মারাত্মক রক্ত স্বল্পতা ছিল
4. আপনার বহুমূত্র রোগ ছিল
5. গর্ভ খালাসের সময় বাচ্চার মাথা আগে আসে নাই
6. নাড়ী আগে খালাস হয়েছিল
7. নাড়ী শিশুর গলায় পেঁচিয়েছিল
8. অতিরিক্ত রক্তপাত
9. প্রসবকালে জ্বর
10. অনিচ্ছা
11. অজানা
12. অন্যান্য ____________________
13. কোন জটিলতা ছিল না

4.1.2 How many months long was the pregnancy?

1. _______ মাস

	কত মাসের গর্ভকাল ছিল?	8. অনিচ্ছা<br>9. অজানা
4.1.3	Was the baby moving in the last few days before the birth?<br>প্রসবের কয়েক দিন আগে থেকে শিশুটি (পেটে) নড়াচড়া করছিল?	1. হ্যাঁ<br>2. না<br>8. অনিচ্ছা<br>9. অজানা
4.1.4	When did you (the mother) last feel the baby move?<br>আপনি (মা) সর্বশেষ কখন বাচ্চার নড়াচড়া অনুভব করতে পেরেছিলেন?	1. প্রসবের _______ দিন আগে<br>2. প্রসবের ________ ঘন্টা আগে<br>8. অনিচ্ছা<br>9. অজানা
4.1.5	Was the liquor foul smelling?<br>তরল পানি গন্ধযুক্ত ছিল?	1. হ্যাঁ<br>2. না<br>8. অনিচ্ছা<br>9. অজানা
4.1.6	Did you (the mother) receive any vaccinations since reaching adulthood including during this pregnancy?<br>প্রাপ্ত বয়স্ক হওয়ার সময় এবং এই গর্ভাবস্থায় (মা) কোন টিকা পেয়েছিলেন কি?	1. হ্যাঁ<br>2. না<br>8. অনিচ্ছা<br>9. অজানা
4.1.7	How many doses?<br>কত ডোজ টিকা নিয়েছিল?	1. এক<br>2. দুই<br>3. তিন<br>4. চার<br>5. পাঁচ বা এর বেশী<br>8. অনিচ্ছা<br>9. অজানা
4.1.8	Was the delivery (How delivery)?<br>শিশুটি কিভাবে ভূমিষ্ঠ হয়েছিল?<br>(উত্তর গুলো পড়ে শুনান এবং একটি চিহ্নিত করুন)	1. নরমাল যন্ত্র প্রয়োগ করে<br>2. নরমাল যন্ত্র ছাড়া<br>3. নরমাল কিন্তু (যন্ত্র/যন্ত্র ছাড়া অজানা)<br>4. সিজারিয়ান অপারেশন<br>8. অনিচ্ছা<br>9. অজানা

5 Neonatal Deaths

5.1	Was the part of the body physically abnormal at time of delivery?<br>প্রসবের পর পর বাচ্চাটির শরীরের কোন অংশ অস্বাভাবিক ছিল? (যেমন, কোন অঙ্গ খুব বড় বা ছোট আকৃতির, শরীরে কোন ফোলা বা চাকা)	1. হ্যাঁ<br>2. না<br>8. অনিচ্ছা<br>9. অজানা
5.2	What were the abnormalities? (Mark all that apply)<br>শিশুর শরীরে অঙ্গ বিকৃতিগুলি কি কি ছিল? (একাধিক উত্তর হতে পারে)<br>(ছবি দেখান)	1. জন্মকালীন অতি ক্ষুদ্রাকৃতির মাথা<br>2. জন্মকালীন অতি বড়াকৃতির মাথা<br>3. মাথার পিছনে বা মেরুদন্ডে মাংশপিন্ড<br>4. অন্যান্য ______________

8. অনিচ্ছা
9. অজানা

5.3 Did the baby breathe immediately after birth?
জন্মের সাথে সাথে শিশুটি শ্বাস নিচ্ছিল?

1. হ্যাঁ
2. না
8. অনিচ্ছা
9. অজানা

5.4 Did the baby have difficulty breathing?
শিশুটির কি শ্বাসকষ্ট হয়েছিল?

1. হ্যাঁ
2. না
8. অনিচ্ছা
9. অজানা

5.5 Was anything done to try to help the baby breathe at birth?
জন্মের সাথে সাথে শ্বাস-প্রশ্বাস চালুর জন্য কোন ব্যবস্থা নেওয়া হয়েছিল?

1. হ্যাঁ
2. না
8. অনিচ্ছা
9. অজানা

5.6 Did the baby cry immediately after birth?
জন্মের সাথে সাথে বাচ্চাটি কি কেঁদেছিল?
যদি হ্যাঁ হয় তবে 5.8 নং প্রশ্নে যান

1. হ্যাঁ
2. না
8. অনিচ্ছা
9. অজানা

5.7 How long after birth did the baby first cry? (Mark One)
জন্মের কতক্ষণ পরে বাচ্চাটি প্রথম কেঁদেছিল? (একটিতে চিহ্ন দিন)

1. ৫ মিনিটের মধ্যে
2. ৬-৩০ মিনিটের মধ্যে
3. ৩০ মিনিটের পরে
4. মোটেও কাঁদে নাই
8. অনিচ্ছা
9. অজানা

5.8 Did the baby stop being able to cry?
বাচ্চাটি কি কাঁদা বন্ধ করে দিয়েছিল?

1. হ্যাঁ
2. না
8. অনিচ্ছা
9. অজানা

5.9 How long before the baby died did the baby stop crying?
মৃত্যুর কতক্ষণ পুর্বে বাচ্চাটির কাঁদা বন্ধ হয়ে গিয়েছিল?

1. এক দিনেরও কম সময়
2. একদিন বা তার বেশী
8. অনিচ্ছা
9. অজানা

5.10 Did the baby ever suckle in a normal way?
বাচ্চাটি কি আদৌ স্বাভাবিকভাবে বুকের দুধ পান করেছিল?

1. হ্যাঁ
2. না
8. অনিচ্ছা
9. অজানা

5.11 During the illness that led to death, did the baby have difficulty breathing?
সর্বশেষ অসুস্থতার সময় শিশুটির কি শ্বাসকষ্ট হয়েছিল?

1. হ্যাঁ
2. না
8. অনিচ্ছা
9. অজানা

5.12 For how many days did the difficult breathing last?
কতদিন শ্বাসকষ্ট ছিল?
(১ দিনের কম হলে = ০০)

1. ______ দিন
8. অনিচ্ছা
9. অজানা

5.13 During the illness that led to death did the baby have fast breathing?
যে অসুখে সে মারা গেল, সেই সময় বাচ্চাটি কি ঘন ঘন শ্বাস ফেলত?

1. হ্যাঁ
2. না
8. অনিচ্ছা
9. অজানা

5.14 For how many days did the fast breathing last?
কতদিন বাচ্চাটির ঘন ঘন শ্বাস ছিল?
(১ দিনের কম হলে = ০০)

1. ______ দিন
8. অনিচ্ছা
9. অজানা

5.15 During the illness that led to death, did the baby have grunting?
যে অসুখে শিশুটি মারা গেল, সেই সময় তার কি গলায় ঘোঁত ঘোঁত শব্দ হতো?

1. হ্যাঁ
2. না
8. অনিচ্ছা
9. অজানা

5.16 During the illness that led to death, did the baby spasms or convulsions?
যে অসুখে সে মারা গেল, সেই সময় শিশুটির খিঁচুনী হয়েছিল কি?

1. হ্যাঁ
2. না
8. অনিচ্ছা
9. অজানা

5.17 During the illness that led to death, did the baby become cold to touch?
যে অসুখে সে মারা গেল, সেই সময় শিশুটির শরীরে হাত দিলে ঠান্ডা অনুভব হতো?

1. হ্যাঁ
2. না
8. অনিচ্ছা
9. অজানা

5.18 During the illness that led to death, did the baby become lethargic after a period normal activity?
যে অসুখে সে মারা গেল, সেই সময় কিছুক্ষণ স্বাভাবিক থাকার পর কি শিশুটির লাই লাই ভাব দেখা যেতো? (Lethergic)

1. হ্যাঁ
2. না
8. অনিচ্ছা
9. অজানা

5.19 During the illness that led to death, did the baby have ulcer(s) (pits)
মৃতুকালীন অসুখের সময় গাঁয়ে কোন ক্ষত ছিল?

1. হ্যাঁ
2. না
8. অনিচ্ছা
9. অজানা

5.20 During the illness that led to death, did he/she have more frequent loose or liquid stools than usual?
যে অসুখে সে মারা গেল, সেই সময় ঘন ঘন পাতলা/তরল পায়খানা হয়েছিল কি?

1. হ্যাঁ
2. না
8. অনিচ্ছা
9. অজানা

5.21 During the illness that led to death, did he/she have yellow skin?
যে অসুখে সে মারা গেল, সেই সময় শিশুটির চামড়ায় হলদে হয়েছিল?

1. হ্যাঁ
2. না
8. অনিচ্ছা
9. অজানা

5.22 Did the infant appear to be healthy and then just die suddenly?
শিশুটি কি সুস্থ দেখাচ্ছিল এবং তারপর হঠাৎ মারা গেল?

1. হ্যাঁ
2. না
8. অনিচ্ছা
9. অজানা

6 Infant & Child Deaths

6.1 During the illness that led to death, did he/she have fever?
যে অসুখে সে মারা গেল সেই সময় কি জ্বর ছিল?

1. হ্যাঁ
2. না
8. অনিচ্ছা
9. অজানা

6.2 How severe was the fever?
জ্বর কতটুকু মারাত্মক ছিল?

1. সামান্য
2. মোটামুটি
3. মারাত্মক
8. অনিচ্ছা
9. অজানা

6.3 During the illness that led to death, did he/she have more frequent loose or liquid stools than usual?
সর্বশেষ যে অসুখে সে মারা গেল, সেই সময় তার কি স্বাভাবিকের চেয়ে ঘন ঘন পাতলা বা তরল পায়খানা হতো?

1. হ্যাঁ
2. না
8. অনিচ্ছা
9. অজানা

6.4 How many stools did she/he have on the day that loose liquid stools were most frequent?
যেদিন পাতলা পায়খানা বেশী ঘন ঘন ছিল সেদিন কতবার পায়খানা হয়েছিল?

1. _______ বার
8. অনিচ্ছা
9. অজানা

6.5 How many days before death did the frequent loose or liquid stools starts?
মৃত্যুর কতদিন আগে থেকে এই ঘন ঘন পাতলা পায়খানা শুরু হয়েছিল?

1. ২৪ ঘন্টার কম সময়
2. _______ দিন
8. অনিচ্ছা
9. অজানা

6.6 Did the frequent loose or liquid stools continue until death?
ঘন ঘন পাতলা পায়খানা কি মৃত্যু পর্যন্ত অব্যাহত ছিল?

1. হ্যাঁ
2. না
8. অনিচ্ছা
9. অজানা

6.7 Was there visible blood in the loose or liquid stools?
পাতলা বা তরল পায়খানার সাথে দৃশ্যত কোন রক্ত ছিল?

1. হ্যাঁ
2. না
8. অনিচ্ছা
9. অজানা

6.8 During the illness that led to death, did the child have a cough?
সর্বশেষ অসুস্থতার সময় শিশুটির কাশি ছিল কি?

1. হ্যাঁ
2. না
8. অনিচ্ছা
9. অজানা

6.9 For how many days did the cough last?

1. _______ দিন

কাশি কতদিন যাবৎ স্থায়ী ছিল?

8. অনিচ্ছা
9. অজানা

6.10 Was the cough very severe?
কাশি কি খুব মারাত্মক ছিল?

1. হ্যাঁ
2. না
8. অনিচ্ছা
9. অজানা

6.11 During the illness that led to death, did ${gen_5_0} have difficult breathing?
সর্বশেষ অসুস্থতার সময় ___________শ্বাসকষ্ট ছিল?

1. হ্যাঁ
2. না
8. অনিচ্ছা
9. অজানা

6.12 For how many days did the difficult breathing last?
শ্বাসকষ্ট কতদিন যাবৎ স্থায়ী ছিল?

1. _______ দিন
8. অনিচ্ছা
9. অজানা

6.13 During the illness that led to death, did ${gen_5_0} have fast breathing?
সর্বশেষ অসুস্থতার সময় কি ঘন ঘন শ্বাস ফেলতো?

1. হ্যাঁ
2. না
8. অনিচ্ছা
9. অজানা

6.14 For how many days did the fast breathing last?
ঘন ঘন শ্বাস ফেলা কতদিন স্থায়ী ছিল?

1. _______ দিন
8. অনিচ্ছা
9. অজানা

6.15 **Grunting**
শ্বাস প্রশ্বাসের সময় ঘোঁত ঘোঁত শব্দ হওয়া

1. হ্যাঁ
2. না
8. অনিচ্ছা
9. অজানা

6.16 Did ${gen_5_0} experience any generalized convulsions or fits during the illness that led to death?
মৃত্যুকালীন অসুস্থতার সময় __________ কোন খিঁচুনী হয়েছিল কিনা?

1. হ্যাঁ
2. না
8. অনিচ্ছা
9. অজানা

6.17 Was she/he unconscious during the illness that led to death?
র্মত্যুকালীন অসুখের সময় সে অজ্ঞান হয়েছিল কিনা?

1. হ্যাঁ
2. না
8. অনিচ্ছা
9. অজানা

6.18 How long before death did unconsciousness start?
মৃত্যুর কতক্ষণ আগে থেকে অজ্ঞান হয়েছিল?

1. ৬ ঘন্টার কম সময়
2. ৬-২৩ ঘন্টার মধ্যে
3. ২৪ ঘন্টা বা তার বেশী
8. অনিচ্ছা
9. অজানা

6.19 Did she/he have a stiff neck during the illness that led to death?
মৃত্যুকালীন অসুস্থতার সময় তার ঘাড় শক্ত হয়ে গিয়েছিল?

1. হ্যাঁ
2. না

8. অনিচ্ছা
9. অজানা

6.20 Did she/he have a bulging fontanelle during the illness that led to death?
মৃত্যুকালীন অসুস্থতার সময় তার মাথার চাঁদি ফুলে গিয়েছিল?

1. হ্যাঁ
2. না
8. অনিচ্ছা
9. অজানা

6.21 During the month before he/she died, did have a skin Rash?
মৃত্যুর আগে ঐ মাসে তার চামড়ায় লালচে দানা উঠেছিল?

1. হ্যাঁ
2. না
8. অনিচ্ছা
9. অজানা

6.22 How many days did the Rash last?
লালচে দানা শরীরে কতদিন স্থায়ী ছিল?

1. _______ দিন
8. অনিচ্ছা
9. অজানা

6.23 During the illness that led to death, did have swollen legs or feet?
মৃত্যুকালীন অসুস্থতার সময় কি তার পা/পায়ের পাতা ফুলে গিয়েছিল?
(ছবি দেখান)

1. হ্যাঁ
2. না
8. অনিচ্ছা
9. অজানা

6.24 How long did the swelling last?
এই ফুলা কতদিন স্থায়ী হয়েছিল?

1. _______ দিন
2. _______ সপ্তাহ
8. অনিচ্ছা
9. অজানা

6.25 During the illness that led to death, did skin flake off in patches?
মৃত্যুকালীন অসুস্থতার সময় তার চামড়া উঠে যেত কিনা?
(Skin flake off in patches)

1. হ্যাঁ
2. না
8. অনিচ্ছা
9. অজানা

6.26 Did his/her hair change in colour to a redish or yellowish colour?
তার চুলের বর্ন বদলে লালচে বা হলদে হয়ে গিয়েছিল?

1. হ্যাঁ
2. না
8. অনিচ্ছা
9. অজানা

6.27 Did his/her have a protruding belly?
তার কি পেট ফুলে গিয়েছিল?

1. হ্যাঁ
2. না
8. অনিচ্ছা
9. অজানা

6.28 During the illness that led to death, did he/she suffer from lack of blood/pallor?
যে অসুখে সে মারা যায় সেই সময় তার রক্ত স্বল্পতা বা ফ্যাকাশে হয়েছিল কি?

1. হ্যাঁ
2. না
8. অনিচ্ছা
9. অজানা

6.29 During the illness that led to death, did he/she have swelling in the armpits?
সর্বশেষ অসুখের সময় তার বগলদ্বয় ফুলে ছিল?

1. হ্যাঁ
2. না

8. অনিচ্ছা
9. অজানা

6.30 During the illness that led to death, did he/she have whitish rash inside the mouth or the tongue?
সর্বশেষ অসুখের সময় তার কি মুখের ভিতর বা জিহবায় Whitish rash হয়েছিল কি?

1. হ্যাঁ
2. না
8. অনিচ্ছা
9. অজানা

6.31 During the illness that led to death, did he/she have bleed from anywhere?
মৃত্যুকালীন অসুখের সময় তার শরীরের কোথাও থেকে রক্তপাত হয়েছিল?

1. হ্যাঁ
2. না
8. অনিচ্ছা
9. অজানা

6.32 During the illness that led to death, did he/she have area of the skin that turned black?
মৃত্যুকালীন অসুখের সময় তার শরীরের চামড়ার কিছু অংশ কালো হয়েছিল?

1. হ্যাঁ
2. না
8. অনিচ্ছা
9. অজানা

7 Health Records

7.1 Was care sought outside the home while she/he had this illness?
মৃত্যুকালীন অসুখের জন্য বাড়ির বাহিরে চিকিৎসা করা হয়েছিল?

1. হ্যাঁ
2. না
8. অনিচ্ছা
9. অজানা

7.2 Where or from whom did you seek care?
কোথায় বা কার নিকট থেকে চিকিৎসা নেয়া হয়েছিল?
(একাধিক উত্তর হতে পারে)

1. Traditional healer
2. হোমিওপ্যাথ
3. আধ্যাত্মিক ব্যক্তিত্ব/ঈমাম
4. সরকারী হাসপাতাল/ICDDR,B
5. সরকারী স্বাস্থ্যকেন্দ্র/ক্লিনিক
6. বেসরকারী হাসপাতাল
7. Community based চিকিৎসক
8. TBA
9. প্রাইভেট চিকিৎসক
10. ফার্মেসী, ঔষধ বিক্রেতা, বাজার
11. অন্যান্য চিকিৎসক
12. আত্মীয়, বন্ধু (বাড়ীর বাহিরে)
13. অনিচ্ছা (উত্তর দানে)
99. অজানা

7.3 Record the name and address of any hospital, health center or clinic where care was sought?
যে কোন হাসপাতাল, স্বাস্থ্যকেন্দ্র বা ক্লিনিকে চিকিৎসা নিয়ে থাকলে প্রতিষ্ঠানের নাম ও ঠিকানা লিখুন।

____

____

7.4 Did a health care worker tell you the cause of death?
স্বাস্থ্যকর্মী/চিকিৎসক কি ওর মৃত্যুর কারণটি বলেছিল?

1. হ্যাঁ
2. না

8. অনিচ্ছা

7.5 What did the health care worker say?
স্বাস্থ্যকর্মী/চিকিৎসক কি কারণটি বলেছিলেন?

7.6 Do you have any health records that belonged to the deceased?
মৃত শিশুর চিকিৎসার কাগজপত্র আছে কি?

7.7 Can I see the health records?
আমাকে এই কাগজপত্র গুলি দেখাবেন?

7.8 Take a picture of the health records.
চিকিৎসার কাগজপত্র গুলি ছবি নিন।

7.9 Are the dates known for the two most recent visit and the last note (Mark all that apply)
সর্বশেষ দুইটি ভিজিটের তারিখ ও শেষ নোট লেখার তারিখ জানার চেষ্টা করুন।

Record the date of the most recent visit
সর্বশেষ ভিজিটের তারিখ তারিখ লিখুন।

দিন মাস বছর

7.10 Record the weight on the most recent visit
সর্বশেষ ভিজিটের সময়কার ওজন লিখুন।

Second most recent visit
দ্বিতীয় সাম্প্রতিক ভিজিট তারিখ

7.11 Record the date of the second most recent visit
দ্বিতীয় সাম্প্রতিক ভিজিটের তারিখ লিখুন।

দিন মাস বছর

7.12 Record the weight on the second most recent visit
দ্বিতীয় সাম্প্রতিক ভিজিটের সময়কার ওজন লিখুন।

7.13 Record the date of the last note
সর্বশেষ ভিজিটের নোটের তারিখ লিখুন।

দিন মাস বছর

7.14 Transcribe the note

7.15 Was a death certificate issuded?
মৃত্যুর প্রত্যায়নপত্র দেওয়া হয়েছিল কিনা?

1. হ্যাঁ
2. না
8. অনিচ্ছা
9. অজানা

7.16 Can I see the death certificate?
আমাকে এই কাগজপত্র গুলি দেখাবেন?

1. হ্যাঁ
2. না

8. অনিচ্ছা
9. অজানা

7.17 Take a picture of the death certificate
চিকিৎসার কাগজপত্র গুলির ছবি নিন।

7.18 Record the immediate cause of death from the certificate
মৃত্যু প্রত্যায়নপত্র থেকে মৃত্যুর তাৎক্ষনিক কারন লিখুন।

7.19 Record the first underlying cause of death from the certificate
প্রথম অর্ন্তনিহিত কারন লিখুন।

7.20 Record the second underlying cause of death from the certificate
দ্বিতীয় অর্ন্তনিহিত কারন লিখুন।

7.21 Record the third underlying cause of death from the certificate
তৃতীয় অর্ন্তনিহিত কারন লিখুন।

7.22 Record the contributing cause(s) of death from the certificate
মৃত্যু প্রত্যায়নপত্র থেকে সহযোগী কারন লিখুন।

7.23 Has the deceased (biological) mother ever been tested for HIV?
মৃত শিশুর মায়ের এইচ আই ভি জীবানুর জন্য পরীক্ষা করা হয়েছিল কি?

1. হ্যাঁ
2. না
8. অনিচ্ছা
9. অজানা

7.24 Was the HIV test ever positive?
এইচ আই ভি জীবানুর পরীক্ষা পজিটিভ ধরা পড়েছিল?

1. হ্যাঁ
2. না
8. অনিচ্ছা
9. অজানা

7.25 Has the deceased's (biological) mother ever been told she had AIDS by a health worker?
কোন স্বাস্থ্য কর্মী কি মাকে বলেছিল যে, মায়ের এইডস রোগ আছে?

1. হ্যাঁ
2. না
8. অনিচ্ছা
9. অজানা

8 Open ended response এবং ইন্টারভিউয়ার এর মন্তব্য/ পর্যবেক্ষন

Instructions: দীর্ঘ সময় যাবৎ বিভিন্ন প্রশ্নের উত্তর দানের জন্য উত্তরদাতাকে ধন্যবাদ জানান এবং এই অসুস্থতা এবং এই মৃত্যু সম্পর্কে নিজের ভাষায় সংক্ষেপে বলার জন্য অনুরোধ করুন।
উত্তরদাতাকে তার নিজের ভাষায় বলার জন্য সহায়তা করুন। উত্তরদাতা বলার পর যতক্ষণ পর্যন্ত না বলে যে, আর কিছু ছিল কিনা? উত্তরদাতার স্বতস্ফূর্তভাবে বলা রোগের লক্ষনগুলো লিপিবদ্ধ করুন ও অপরিচিত কোন শব্দ থাকলে তার নীচে লাইন টেনে চিহ্নিত করুন।

POPULATION HEALTH METRICS RESEARCH CONSORTIUM
VERBAL AUTOPSY GENERAL INFORMATION MODULE

1 Consent Section

Interviewer: উত্তরদাতাকে অনুমতি পত্রটি পড়ে শুনান। উত্তরদাতার কোন প্রশ্ন আছে কিনা জিজ্ঞাসা করুন। উত্তরদাতার প্রশ্নের জবাবদানের পর তাকে এই কার্যক্রমে অংশ নিতে সম্মতি আছে কিনা জিজ্ঞাসা করম্নন।

1.1 Take an image of the signed consent form.

1.2 Scan the barode of the paper consent form.

1.3 Record Video of respondent acknowledging consent

1.4 Record audio of respondent acknowledging consent

1.5 Did respondent give consent?

1. হ্যাঁ
2. না
9. অজানা

No consent

Thanks the respondent for his/her time and end the interview.
সময় দেওয়ার জন্য তাকে ধন্যবাদ জানান এবং সাক্ষাৎকার এখানেই শেষ করুন।

2 মৃত ব্যক্তির প্রয়োজনীয় তথ্যাবলী

জন্ম তারিখ

দিন		মাস		বছর			

2.1 What was the name of deceased?
মৃত ব্যক্তির নাম: ____

2.2 What was the sex of the deceased?
মৃতের লিঙ্গা:

পুরুষ ☐ মহিলা ☐

মৃত্যুর তারিখঃ

দিন		মাস		বছর			

2.3 What was the last known age of the deceased?
মৃতের বয়সঃ

(১ দিনের কম হলে ০০ লিখুন)
99 = অজানা

		বছর (১ বছর বা তার বেশী)
		মাস (১ বছরের কম হলে)
		দিন (১ মাসের কম হলে)

3 Injuries and Accidents section
আঘাত/দুর্ঘটনা

3.1 Did ___ suffer from any injury or accident that led to his/her death?
______________ এরকম কোন আঘাত বা দুর্ঘটনায় পতিত হয়ে ছিলেন কি?

1. হ্যাঁ
2. না
8. অনিচ্ছা
9. অজানা

What kind of injury or accident did suffer from? (Read through the list in sequence and MARK ALL THAT APPLY)
কি ধরনের আঘাত বা দুর্ঘটনা হয়েছিল তা পড়ে শুনান এবং যেটি/যেগুলি প্রযোজ্য সেটি চিহ্নিত করুন।

1. সড়ক দুর্ঘটনা
2. পড়ে যাওয়া
3. পানিতে ডুবে
4. বিষক্রিয়া
5. বিষাক্ত প্রানীর কামড়
6. পুড়ে যাওয়া
7. অপমৃত্যু (আত্মহত্যা, হত্যা, নির্যাতন)
8. অনিচ্ছা
9. অজানা
10. অন্যান্য আঘাত

3.2 What kind of injury or accident did he/she suffer from?
কি ধরনের আঘাত বা দুর্ঘটনা হয়েছিল তা পড়ে শুনান।

1. সড়ক দুর্ঘটনা
2. পড়ে যাওয়া
3. পানিতে ডুবে
4. বিষক্রিয়া
5. বিষাক্ত প্রানীর কামড়
6. পুড়ে যাওয়া
7. অপমৃত্যু (আত্মহত্যা, হত্যা, নির্যাতন)
8. অনিচ্ছা
9. অজানা
10. অন্যান্য আঘাত

3.3 Was the injury or accident self-inflicated?
এই আঘাত বা দুর্ঘটনাটি কি নিজের ইচ্ছায় সংঘটিত হয়েছিল?

1. হ্যাঁ
2. না
8. অনিচ্ছা
9. অজানা

3.4 Was the injury or accident intentionally inflicated by someone else?
এই আঘাত বা দুর্ঘটনাটি কি অন্যের দ্বারা সংঘটিত হয়েছিল?

1. হ্যাঁ
2. না
8. অনিচ্ছা
9. অজানা

4 মৃত ব্যক্তির নিন্মলিখিত অসুখগুলির কোনটি ছিল কিনা?

4.1 হাঁপানী (Asthma)

1. হ্যাঁ
2. না
8. অনিচ্ছা
9. অজানা

স্ট্রোক (Stroke)

1. হ্যাঁ
2. না
8. অনিচ্ছা
9. অজানা

যক্ষা (Tuberculosis)

1. হ্যাঁ
2. না
8. অনিচ্ছা
9. অজানা

এইডস (AIDS)

1. হ্যাঁ
2. না
8. অনিচ্ছা
9. অজানা

ক্যানসার (Cancer)

1. হ্যাঁ
2. না
8. অনিচ্ছা
9. অজানা

COPD (Chronic Obstructive Pulmonary Disease)

1. হ্যাঁ
2. না
8. অনিচ্ছা
9. অজানা

বহুমুত্র রোগ (Diabetes)

1. হ্যাঁ
2. না
8. অনিচ্ছা
9. অজানা

মৃগী রোগ (Epilepsy)

1. হ্যাঁ
2. না
8. অনিচ্ছা
9. অজানা

ü` রোগ (Heart Disease)

1. হ্যাঁ
2. না
8. অনিচ্ছা
9. অজানা

5 Symptom checklist section

5.1 For how long was ill before she/he died?
মৃত্যুর কতদিন আগে থেকে উনি অসুস্থ ছিলেন?

1. _______ মাস
2. _______ দিন
8. অনিচ্ছা
9. অজানা

5.2 Did have Rash?
চামড়ায় লালচে দানা ছিল?

1. হ্যাঁ
2. না
8. অনিচ্ছা
9. অজানা

5.3 How many days did have the Rash?
কত দিন যাবৎ ______________গায়ে লালচে দানা ছিল?

1. __________ দিন
8. অনিচ্ছা
9. অজানা

5.4 Where was the Rash located?
শরীরের কোন কোন জায়গায় লালচে দানা ছিল?

1. মুখে
2. পেটে, পিঠে
3. হাতে, পায়ে
4. সারা গাঁয়ে
5. অন্যান্য
8. অনিচ্ছা
9. অজানা

5.5 Did have Sores?
____________কি গায়ে ক্ষত ছিল (Sores)?

1. হ্যাঁ
2. না
8. অনিচ্ছা
9. অজানা

5.6 Did the sores have clear fluid or pus?
ক্ষত স্বচ্ছ পানিযুক্ত বা পুঁজযুক্ত ছিল কি?

1. হ্যাঁ
2. না
8. অনিচ্ছা
9. অজানা

5.7 Did have an Ulcer (Pit) on foot?
___________ পাঁয়ে কোন ঘা ছিল কি?

1. হ্যাঁ
2. না
8. অনিচ্ছা
9. অজানা

5.8 Did the Ulcer Ooze pus?
এই ঘাঁ থেকে পুঁজ বের হতো কি?

1. হ্যাঁ
2. না
8. অনিচ্ছা
9. অজানা

5.9 For how many days did the Ulcer Ooze pus?
কত দিন যাবৎ ঘাঁ থেকে পুঁজ বের হতো?

1. __________ দিন
8. অনিচ্ছা
9. অজানা

5.10 Did have yellow discolouration of the eyes?
______________ চক্ষুর বর্ন হলদে হয়ে গিয়েছিল?

1. হ্যাঁ
2. না
8. অনিচ্ছা
9. অজানা

5.11 For how long did have the yellow discolouration?
মৃত্যুর কতদিন আগে থেকে চক্ষু হলদে হয়েছিল?

1. __________ মাস
2. __________ দিন
8. অনিচ্ছা
9. অজানা

5.12 Did have puffiness of the face?
_______________ মুখ ফুলে গিয়েছিল কি?

1. হ্যাঁ
2. না
8. অনিচ্ছা
9. অজানা

5.13 For how long did have puffiness of the face?
_______________ এই মুখ ফোলা কত দিন যাবৎ ছিল?

1. __________ দিন
2. __________ মাস
8. অনিচ্ছা
9. অজানা

5.14 Did have general puffiness all over his/her body?
_______________ সারা শরীর ফুলে গিয়েছিল কি?

1. হ্যাঁ
2. না
8. অনিচ্ছা
9. অজানা

5.15 Did have a Lump in the neck?
_______________ ঘাড়ে কোন চাকা ছিল কি?

1. হ্যাঁ
2. না
8. অনিচ্ছা
9. অজানা

5.16 Did have a lump in the armpit?
______________ বগলে কোন চাকা ছিল কি?

1. হ্যাঁ
2. না
8. অনিচ্ছা
9. অজানা

5.17 Did have a lump in the groin?
______________ কুঁচকিতে কোন চাকা ছিল কি?

1. হ্যাঁ
2. না
8. অনিচ্ছা
9. অজানা

5.18 Did have a cough?
_______________ কাঁশি ছিল কি?

1. হ্যাঁ
2. না
8. অনিচ্ছা
9. অজানা

5.19 For how long did have a cough?
______________ কতদিন যাবৎ কাঁশি ছিল?

1. __________ মাস
2. __________ দিন
8. অনিচ্ছা
9. অজানা

5.20 Did the cough produce sputum?
কাঁশির সময় কি কফ বের হতো?

1. হ্যাঁ
2. না
8. অনিচ্ছা
9. অজানা

5.21 Did cough blood?
______________ কাঁশির সাথে কি রক্ত যেত?

1. হ্যাঁ
2. না
8. অনিচ্ছা
9. অজানা

5.22 Did have difficulty breathing?
_____________ শ্বাসকষ্ট ছিল কি?

1. হ্যাঁ
2. না
8. অনিচ্ছা
9. অজানা

5.23 Was the difficulty continous or on and off?
এই শ্বাসকষ্ট কি একটানা হত নাকি মাঝে মাঝে হত?

1. একটানা
2. মাঝে মাঝে
8. অনিচ্ছা
9. অজানা

5.24 Did he/she experience pain in the chest in the month preceding death?
মৃত্যুর আগে ঐ মাসে _______________ বুকে ব্যথা ছিল কিনা?

1. হ্যাঁ
2. না
8. অনিচ্ছা
9. অজানা

5.25 How long did the pain last?
কতক্ষণ যাবৎ এই বুক ব্যথা থাকত?

1. ৩০ মিনিটের কম সময়
2. ৩০ মিনিট থেকে ২৪ ঘন্টা
3. ২৪ ঘন্টার বেশী
8. অনিচ্ছা
9. অজানা

5.26 Did have more frequent loose or liquid stools then usual?
স্বাভাবিকের তুলনায় ___________ কি ঘন ঘন পাতলা বা তরল পায়খানা হতো?

1. হ্যাঁ
2. না
8. অনিচ্ছা
9. অজানা

5.27 Was there blood in the stool?
তার কি পায়খানার সাথে রক্ত যেত?
উত্তর যদি না, অজানা বা অনিচ্ছা হয় 2.52 নং পশ্নে যান।

1. হ্যাঁ
2. না
8. অনিচ্ছা
9. অজানা

5.28 Was there blood in the stool up until death?
পায়খানার সাথে রক্ত যাওয়া কি মৃত্যুর পুর্ব পর্যন্ত ছিল?

1. হ্যাঁ
2. না
8. অনিচ্ছা
9. অজানা

5.29 Did stop urinating?
মৃত্যুর আগে ____________ প্রস্রাব বন্ধ হয়ে গিয়েছিল?

1. হ্যাঁ
2. না
8. অনিচ্ছা
9. অজানা

5.30 Did vomit in the week preceding the death?
মৃত্যুর আগের সপ্তাহে __________ বমি হতো?

1. হ্যাঁ
2. না
8. অনিচ্ছা
9. অজানা

5.31 Was there blood in the vomit?
বমির সাথে রক্ত গিয়েছিল?

1. হ্যাঁ
2. না
8. অনিচ্ছা
9. অজানা

5.32 Was the vomit black?
বমির রং কি কালো ছিল?

1. হ্যাঁ
2. না
8. অনিচ্ছা
9. অজানা

5.33 Did have difficulty swallowing?
____________ কি খাবার গলাধঃকরনে বা গিলতে অসুবিধা হয়েছিল?

1. হ্যাঁ
2. না
8. অনিচ্ছা
9. অজানা

5.34 For how long before death did have difficulty swallowing?
মৃত্যুর কতদিন আগে থেকে গলাধঃকরনে বা গিলতে অসুবিধা ছিল?

1. __________ মাস
2. __________ দিন
8. অনিচ্ছা
9. অজানা

5.35 Was the difficulty with swallowing with solids, liquid, or both?
গলাধঃকরনে এই অসুবিধা কি শক্ত খাবার বা তরল খাবার নাকি উভয় খাবারের সময়ই ছিল?

1. শক্ত খাবার
2. তরল
3. উভয় খাবার
8. অনিচ্ছা
9. অজানা

5.36 Did have pain upon swallowing?
গলাধঃকরন বা গিলতে _____________ ব্যথা হতো কি?

1. হ্যাঁ
2. না
8. অনিচ্ছা
9. অজানা

5.37 Did have belly pain?
______________ পেট ব্যথা ছিল কি?

1. হ্যাঁ
2. না
8. অনিচ্ছা
9. অজানা

5.38 For how long before death did have belly pain?
কতক্ষণ যাবৎ ______________ পেটব্যথা ছিল?

1. __________ ঘন্টা
2. __________ দিন
3. __________ মাস
8. অনিচ্ছা
9. অজানা

5.39 Was the pain in the uper or lower belly?
এই ব্যথাটি কি পেটের উপর দিকে না নীচের দিকে ছিল?

1. উপর পেটে
2. নীচ পেটে
8. অনিচ্ছা
9. অজানা

5.40 Did have a more then protruding belly?
স্বাভাবিকের চেয়ে _____________ পেট ফুলে গিয়েছিল?

1. হ্যাঁ
2. না
8. অনিচ্ছা
9. অজানা

5.41 How rapidly did develop the protruding belly?
_____________ পেট যে ফুলেছিল, তা কত দ্রুত হয়েছিল?

1. দ্রুত
2. ধীরে ধীরে
8. অনিচ্ছা
9. অজানা

5.42 Did have any mass in the belly?
_______________ পেটে কোন চাকা ছিল?

1. হ্যাঁ
2. না
8. অনিচ্ছা
9. অজানা

5.43 For how long before death did have a mass in the belly?
মৃত্যুর কতদিন আগে থেকে পেটে চাকা ছিল?

1. __________ মাস
2. __________ দিন
8. অনিচ্ছা
9. অজানা

5.44 Did have a stiff neck?
______________ কি ঘাড় শক্ত হয়ে গিয়েছিল?

1. হ্যাঁ
2. না
8. অনিচ্ছা
9. অজানা

5.45 For how long before death did have stiff neck?
মৃত্যুর কতদিন আগে ______________ ঘাড় শক্ত হয়ে গিয়েছিল?

1. __________ মাস
2. __________ দিন
8. অনিচ্ছা
9. অজানা

5.46 Did experience a period of loss consciousness?
______________ কি কখনো অজ্ঞান হয়ে গিয়েছিল?

1. হ্যাঁ
2. না
8. অনিচ্ছা
9. অজানা

5.47 Did the period of loss conciousness start suddenly or slowly?
অজ্ঞান যে হয়েছিল সেটি কি দ্রুত না ধীরে ধীরে শুরু হয়েছিল?

1. দ্রুত
2. ধীরে ধীরে
8. অনিচ্ছা
9. অজানা

5.48 Did it continue until death?
এই অজ্ঞানাবস্থা কি মৃত্যু পর্যন্ত ছিল?

1. হ্যাঁ
2. না
8. অনিচ্ছা
9. অজানা

5.49 Did have convulsions?
খিঁচুনী হয়েছিল?
(Demonstrate)

1. হ্যাঁ
2. না
8. অনিচ্ছা
9. অজানা

5.50 For how long before death did the convulsions last?
মৃত্যুর পুর্বে খিঁচুনী কতক্ষণ স্থায়ী ছিল?

1. __________ মিনিট
2. __________ ঘন্টা
8. অনিচ্ছা
9. অজানা

5.51 Did the person become unconcious immediately after the convulsions?
খিঁচুনীর পর পর সে কি অজ্ঞান হয়ে গিয়েছিল?

1. হ্যাঁ
2. না
8. অনিচ্ছা
9. অজানা

5.52 Was in any part of the body paralyzed?
_____________ শরীরের কোন অংশ অবশ হয়ে গিয়েছিল?

1. হ্যাঁ
2. না
8. অনিচ্ছা
9. অজানা

5.53 Which were the limbs or body parts paralyzed (Read through the list in sequence and mark that all apply)
শরীরের কোন কোন অঙ্গ প্রত্যঙ্গ অবশ হয়ে গিয়েছিল?
(তালিকাটি পড়ে শুনান, একাধিক উত্তর চিহ্নিত হতে পারে)

1. ডান হাত/ডান পা
2. বাম হাত/বাম পা
3. শরীরের নীচের অংশ
4. শরীরের উপরের অংশ
5. শুধু এক পা
6. শুধু এক হাত
7. সারা শরীর
8. অনিচ্ছা
9. অজানা
10. অন্যান্য _____________

6 Questions for women section

6.1 Did have any swelling or lump in the breast?
____________ স্তনে কোন ফোলা বা চাকা ছিল কি?

1. হ্যাঁ
2. না
8. অনিচ্ছা
9. অজানা

6.2 Did have any Ulcers (Pits) in the breast?
_____________ স্তনে কোন ঘাঁ ছিল কি?

1. হ্যাঁ
2. না
8. অনিচ্ছা
9. অজানা

6.3 Did have Vaginal bleeding after cessation of menstruations? (Post menopausal)
মাসিক একেবারে বন্ধ হয়ে যাওয়ার পর আবার রক্তপাত হয়েছিল কি?

1. হ্যাঁ
2. না
8. অনিচ্ছা
9. অজানা

6.4 Did have Vaginal bleeding other than her period? (Intramenostrual)
দুই মাসিকের মধ্যবর্তী সময়ে রক্তক্ষরণ হতো কি?

1. হ্যাঁ
2. না
8. অনিচ্ছা
9. অজানা

6.5 Was there excessive Vaginal bleeding in the week prior to death?
মৃত্যুর আগে ঐ এক সপ্তাহে অতিরিক্ত রক্তক্ষরণ হয়েছিল কি?

1. হ্যাঁ
2. না
8. অনিচ্ছা
9. অজানা

6.6 At that time was her period over due?
মৃত্যুর সময় উনার মাসিকের সময় পার হয়ে গিয়েছিল কি?

1. হ্যাঁ
2. না
8. অনিচ্ছা
9. অজানা

6.7 If overdue, for how many weaks?
যদি হয়ে থাকে তাহলে কত সপ্তাহ পার হয়ে গিয়েছিল?

1. __________ সপ্তাহ
8. অনিচ্ছা
9. অজানা

6.8 Did have a shapain in the belly shortly before death?
মৃত্যুর ঠিক পুর্বে পেটে তীব্র ব্যথা ছিল কি?

1. হ্যাঁ
2. না
8. অনিচ্ছা
9. অজানা

6.9 Was pregnant at time of death?
মৃত্যুর সময় গর্ভবতী ছিলেন?

1. হ্যাঁ
2. না
8. অনিচ্ছা
9. অজানা

6.10 For how many months was she pregnant?
গর্ভবতী হয়ে থাকলে তিনি কত মাসের গর্ভবতী ছিলেন?

1. __________ মাস
8. অনিচ্ছা
9. অজানা

6.11 Did she die during an abortion?
উনি কি গর্ভপাত হওয়ার সময় মারা গিয়েছিলেন?

1. হ্যাঁ
2. না
8. অনিচ্ছা
9. অজানা

6.12 Did bleeding occur while she was pregnant?
উনি যখন গর্ভবতী ছিলেন তখন কি রক্তপাত হয়েছিল?

1. হ্যাঁ
2. না
8. অনিচ্ছা
9. অজানা

6.13 Did she have exessive bleeding during labour or delivery?
প্রসব ব্যথার বা গর্ভখালাসের সময় উনার অতিরিক্ত রক্তপাত হয়েছিল কি?

1. হ্যাঁ
2. না
8. অনিচ্ছা
9. অজানা

6.14 Did she die during labour or delivery?
উনি কি প্রসব ব্যথা চলাকালীন/প্রসবের সময় (During labor or delivery) মৃত্যুবরন করেছিলেন (প্রসব ব্যথা তখনই বলা যাবে যখন এই ব্যথা ১০ মিনিটের মধ্যে বার বার হবে।)

1. হ্যাঁ
2. না
8. অনিচ্ছা
9. অজানা

6.15 For how many hours was she in labour?
উনার প্রসব ব্যথা কতক্ষণ ছিণ?
প্রসব ব্যথার সময় উনি মারা গেলে সেকশন 4 এ যান)

1. __________ ঘন্টা
8. অনিচ্ছা
9. অজানা

6.16 Did she die within six weeks of having an abortion?
উনি কি গর্ভপাত হওয়ার ৬ সপ্তাহের মধ্যে মারা গিয়েছিলেন?

1. হ্যাঁ
2. না
8. অনিচ্ছা
9. অজানা

6.17 Did she die withing six weeks or child birth?
উনি কি বাচ্চা প্রসবের ৬ সপ্তাহ সময়ের মধ্যেই মারা গিয়েছিল?

1. হ্যাঁ
2. না
8. অনিচ্ছা
9. অজানা

6.18 Did she have excessive bleeding after delivery or abortion?
উনার গর্ভখালাশের /গর্ভপাতের পর অতিরিক্ত রক্তপাত হয়েছিল কি?

1. হ্যাঁ
2. না
8. অনিচ্ছা
9. অজানা

7 Tobacco Section

7.1 Did use Tocacco?
______________ ধুমপান করতেন?
(সিগারেট, বিড়ি, জর্দাসহ পান খাওয়ার অভ্যাস থাকলে)
উত্তর যদি না, অজানা বা অনিচ্ছা হয় 4.5 নং প্রশ্নে যান ।

1. হ্যাঁ
2. না
8. অনিচ্ছা
9. অজানা

7.2 What kind of Tobacco did he/she use?
______________ কি ধরনের ধুমপান করতেন?

1. সিগারেট
2. পাইপ
3. গুল (Chewing tobacco)
4. বিড়ি (Local form of tobacco)
5. অন্যান্য ______________
8. অনিচ্ছা _______
9. অজানা

7.3 How many Cigarettes/Biri did smoke daily?
_____________ দিনে কতবার সিগারেট সেবন ব্যবহার করতেন?

1. __________ বার
8. অনিচ্ছা
9. অজানা

8 Health Records Section (স্বাস্থ্য সম্পর্কিত তথ্য)

8.1 Was care sought outside the home while had this illness?
অসুস্থকালীন সময়ে চিকিৎসার জন্য _______________কোথাও নেয়া হয়েছিল?
উত্তর যদি না, অজানা বা অনিচ্ছা হয় 6.4 নং প্রশ্নে যান ।

1. হ্যাঁ
2. না
8. অনিচ্ছা
9. অজানা

8.2 Where or from home did you seek care? (Check all that apply)
কোথায় বা কার নিকট থেকে চিকিৎসা নেয়া হয়েছিল?
(একাধিক উত্তর হতে পারে)

1=Indegenous healer, 2=Homeopath, 3=Spiritual healer, 4= Government hospital/icddr,b
5=Government health centre/clinic, 6=Private hospital, 7- Community physician,
8=TBA, 9= Private doctor, 10=Phermacy/drug seller, 11= other practioner,
12= Relative/friend (outside home), 13=refuse to tell, 99= unknown

1. সনাতনী চিকিৎসা 2. হোমিওপ্যাথ 3. ধর্মীয় নেতা/ঈমাম 4. সরকারী হাসপাতাল/ICDDR,B
5. সরকারী স্বাস্থ্য কেন্দ্র/ক্লিনিক 6. প্রাইভেট হাসপাতাল 7. কম্যুনিটি চিকিৎসক
8. টি বি এ 9. প্রাইভেট চিকিৎসক 10. ফার্মেসী, ঔষধ বিক্রেতা, দোকান
8.3 11. অন্যান্য চিকিৎসক 12. আত্মীয়, বন্ধু (বাড়ির বাহিরে) 13. অনিচ্ছা 99. অজানা

Record the name of address of any hospital, health centre or clinic where care was sought?
হাসপাতাল, স্বাস্থ্যকেন্দ্র নিয়ে চিকিৎসা করালে প্রতিষ্ঠানের নাম ও ঠিকানা লিখুন।

8.4

Did a health care worker tell you the cause of death?
চিকিৎসক/মেডিকেল ষ্টাফ আপনাদেরকে উনার মৃত্যুর কারণ বলেছিলেন?
মৃত ব্যক্তির চিকিৎসার কোন কাগজ-পত্র আছে কি?

1. হ্যাঁ
2. না
8. অনিচ্ছা
9. অজানা

8.5

What did the health care worker say?
উনি কি কারণটি বলেছিলেন?

8.6

Where did the deceased die?
মৃত ব্যক্তি কোথায় মৃত্যবরন করেছিলেন?

Where did the deceased die? [Other, specified]
অন্যান্য স্থানে হলে নির্দিষ্ট করে লিখুন।

8.7

Do you have any health records that belonged to the deceased?
মৃত ব্যক্তির চিকিৎসার কোন কাগজ-পত্র আছে কি?

1. হ্যাঁ
2. না
8. অনিচ্ছা
9. অজানা

8.8

Can I see the health records?
আমি কি চিকিৎসার কাগজ-পত্র দেখতে পারি?
উত্তর যদি না, অজানা বা অনিচ্ছা হয় 6.9 নং প্রশ্নে যান । উত্তর যদি হ্যাঁ হয় তাহ হলে তথ্যগুলো লিখে নিন।

1. হ্যাঁ
2. না
8. অনিচ্ছা
9. অজানা

8.9

Take a picture of the health records.
চিকিৎসার কাগজপত্র গুলির একটি ছবি নিন।

8.10

Are the dates known for the most recent visits and the last notes? (Mark all that apply)
সর্বশেষ দুইটি ভিজিটের তারিখ এবং শেষ ভিজিটের নোটটি লিখুন।

Most recent visit

দিন মাস বছর

8.11

2nd most recent visit

দিন মাস বছর

8.12

Last note

দিন মাস বছর

সর্বশেষ নোট লিখার তারিখটি লিখুন।

8.13

Transcribe the note

এখানে নোটটি লিখুন।

8.14 ______________________________ 1. হ্যাঁ

Was a death certificate issued? 2. না

উনার কি মৃত্যু প্রত্যায়নপত্র (Death Certificate) আছে? 8. অনিচ্ছা

9. অজানা

8.15

Can I see the death Certificate? 1. হ্যাঁ

আমি কি মৃত্যু প্রত্যায়নপত্র দেখতে পারি? 2. না

8. অনিচ্ছা

9. অজানা

8.16

Take a picture of the death certificate.

মৃত্যুর প্রত্যায়নপত্রের ছবি নিন।

Mark all cause of death information that is known.

মৃত্যুর কারণ সমুহ যা জানা যায় লিখুন।

8.17

Record the immidiate cause of death from the certificate

সার্টিফিকেট থেকে মৃত্যর তাৎক্ষনিক কারণটি লিখুন।

8.18

Record the first underlying cause of death from the certificate

সার্টিফিকেট থেকে মৃত্যর প্রথম অর্ন্তনিহিত কারণ লিখুন।

8.19

Record the second underlying cause of death from the certificate

সার্টিফিকেট থেকে মৃত্যুর দ্বিতীয় অর্ন্তনিহিত কারনটি লিখুন।

8.20

Record the third underlying cause of death from the certificate

সার্টিফিকেট থেকে মৃত্যুর তৃতীয় অর্ন্তনিহিত কারনটি লিখুন।

8.21

Record the contributing cause(s) of death from the certificate

সার্টিফিকেট থেকে মৃত্যুর সহযোগী কারন লিখুন।

9

Open Ended Response and Interviewer Comments/Observation Section

Instructions to the interviewer: Say to the respondent: "Thank you for telling me all about ${gen_5_0}'s final illness. We have asked you some very detailed questions. Would you now like to tell me in your own words the story of the illness that led to ${gen_5_0}'s death and anything else you feel that should be included."

To the interviewer: Write down what the respondent tells you in his/her own words. Do not prompt except for asking whether there was anything else after the respondent finishes. While recording, indicate any unfamiliar terms. You may also use this space to write down your comments and observations about the interview.

9.1

Open-ended response and interviewer comments

SECTION 1: BACKGROUND

1.1.	Gipanganak ba siyang usa ra o duna siya'y kaluha ?	1	Singleton	|___|
		2	Multiple	|___|
		8	Refuses to answer	|___|
		9	Don't know	|___|

IF TWO OR MORE CHILDREN ARE BORN AT THE SAME TIME, IT IS COUNTED AS A MULTIPLE BIRTH, EVEN IF ONE OR MORE OF THE BABIES ARE BORN DEAD.

IF 1.1. IS "SINGLETON", SKIP TO 1.3.

1.2.	Ikapila man ni siya sa mga anak nimo – una, ika-duha, Etc.?	1	First	|___|
		2	Second	|___|
		3	Third or more	|___|
		8	Refuses to answer	|___|
		9	Don't know	|___|

IF MOTHER IS PRESENT, GO TO 1.6. IF MOTHER IS NOT PRESENT AT THE INTERVIEW, ASK :

1.3.	Buhi pa ba ang ilang inahan? **IF YES, GO TO 1.6.**	1	Yes	|___|
		2	No	|___|
1.4.	Namatay ba inahan sa pagpa-nganak o paghuman sa pag-panganak niya ? **IF DURING DELIVERY GO TO 1.6.**	1	During	|___|
		2	After	|___|
		8	Refuses to answer	|___|
		9	Don't know	|___|
1.5.	Kanus-a man namatay ang Inahan ? **LESS THAN 24 HRS = 00 DAYS USE 1 MONTH = 30 DAYS TO DETERMINE THE NUMBER OF MONTHS.**	1	|___||___| days **OR**	
		2	|___||___| months	
		8	Refuses to answer	|___|
		9	Don't know	|___|

1.6.	Diin man namatay ang bata?	1	Hospital	|___|
		2	Other health facility	|___|
		3	En route to hospital or other health facility	|___|
		4	Home	|___|
		5	Other _____________	|___|
		8	Refuses to answer	|___|
		9	Don't know	|___|
1.7.	Unsa man ang gidak-on sa Bata adtong gi-anak siya.. **READ THE QUESTION AND SLOWLY READ THE FIRST 4 CHOICES. RESPONDENT HEAR ALL 4 CHOICES AND THEN RESPOND. SHOW PHOTOS**	1	Very small	|___|
		2	Smaller than usual	|___|
		3	About average	|___|
		4	Larger than usual	|___|
		8	Refuses to answer	|___|
		9	Don't know	|___|
1.8..	Unsa man ang timbang sa bata pagkatawo niya ?	1	|___|. |___| pounds	
		8	Refuses to answer	|___|
		9	Don't know	|___|
1.9.	Lalaki o babaye ba kadto siya?	1	Male	|___|
		2	Female	|___|
		8	Refuses to answer	|___|
		9	Don't know	|___|
1.10.	Unsa man ang petsa sa iyang pagkatawo ?	1	|___|___|/|___|___|/|___|___|___|___| Mm dd yyyy	
		8	Refuses to answer	|___|
		9	Don't know	|___|
1.11.	Buhi o patay ba ang bata pagkatawo niya ?	1	Alive	|___|
		2	Dead	|___|
		8	Refuses to answer	|___|
		9	Don't know	|___|

1.12.	Nihilak ba ang bata pagka-tawo niya ?	1	Yes	|___|
		2	No	|___|
		8	Refuses to answer	|___|
		9	Don't know	|___|
1.13.	Nilihok ba ang bata pagka-tawo niya?	1	Yes	|___|
		2	No	|___|
		8	Refuses to answer	|___|
		9	Don't know	|___|
1.14.	Niginhawa ba ang bata pagkatawo niya ?	1	Yes	|___|
		2	No	|___|
		8	Refuses to answer	|___|
		9	Don't know	|___|

1.15. **INTERVIEWER ONLY**

REFER TO QUESTIONS 1.12., 1.13., AND 1.14. IF ALL THREE RESPONSES ARE "NO" THEN CHECK "YES". OTHERWISE, CHECK "NO."

YES |___| NO |___|
STOP

IF YOU ANSWERED "YES" TO 1.15 (STILLBIRTH) GO TO 1.16.

IF YOU ANSWERED "NO" TO 1.15 (LIVEBIRTH) GO TO 1.20

1.16.	Diha bay mga samad-samad o timalhan nga nabun-og ang bata pagkatawo?	1	Yes	|___|
		2	No	|___|
		8	Refuses to answer	|___|
		9	Don't know	|___|
1.17.	Kunot ba kayo ang panit sa bata ?	1	Yes	|___|
		2	No	|___|
		8	Refuses to answer	|___|
		9	Don't know	|___|
1.18.	Diha bay parte sa lawas sa bata nga dili normal adtong pagkatawo niya? (pananglitan sobra kadako, sobra ka gamay o dunay nitubo sa lawas.	1	Yes	|___|
		2	No	|___|
		8	Refuses to answer	|___|
		9	Don't know	|___|

IF "NO" OR "DON'T KNOW" OR 'REFUSES TO ANSWER" GO TO SECTION 2.

1.19.	Unsa man ang dili normal ?	1	Head size very small at the time of birth ?	|___|
	MARK ALL THAT APPLY.	2	Head size very large at time of birth?	|___|
		3	Mass defect on the back of the head or spine	|___|
		4	Others, specify	|___|
			_________________	|___|
		8	Refuses to answer	|___|

STOP

AFTER COMPLETING 1.19., CONTINUE TO SECTION 2 MATERNAL HISTORY.

1.20.	Pila man ang edad sa bata dihang nagsugod ang iyang sakit nga nakaingon sa iyang pagkamatay? **Less than 24 hours = 00 days** **Use 1 month = 28 days to determine the number of months)**	1	|___||___| days (if less than one month)
		2	|___||___| Months (if less than one year)
		3	|___||___| Years (one year or older)
		8	Refuses to answer |___|
		9	Don't know |___|

1.21. Unsa man kadugay ang iyang sakit ?
Less than 24 hours = 00 days
Use 1 month = 30 days to determine the number of months)

1 |___||___| days **OR**
2 |___||___| months
8 Refuses to answer |___|
9 Don't know |___|

1.22. Diin man namatay ang bata?

1 Hospital |___|
2 Other health facility |___|
3 En route to hospital Or health facility |___|
4 Home |___|
5 Others, specify |___|
____
8 Refuses to answer |___|
9 Don't know |___|

1.23. **FOR DEATHS AT A HOSPITAL OR A HEALTH FACILITY, RECORD FACILITY NAME AND ADDRESS**

1.24 Unsa man ang petsa sa iyang pagkamatay ?

1 |___|___|/|___|___|/|___|___|___|___|
Mm dd yyyy
8 Refuses to answer |___|
9 Don't know |___|

1.25. Pila man ang iyang edad adtong namatay siya ?
Use 1 month = 28 days to determine the number of months)

1 |___||___| days (if less than one month)
2 |___||___| Months (if less than one year)
3 |___||___| Years (one year or older)
8 Refuses to answer |___|
9 Don't know |___|

1.26. **MARK THE BABY'S AGE AT THE TIME OF DEATH.**

1 Less than 28 days old |___|
2 28 days – 11 years |___|

INTERVIEWER :

IF 1.25 IS "REFUSES TO ANSWER" OR "DON'T KNOW", USE YOUR BEST JUSDGMENT TO ANSWER 1.26.

STOP

If the child is less than 28 days old, CONTINUE TO SECTION 2: MATERNAL HISTORY.

If the child is 28 days to 11 years old, GO TO SECTION 4: INFANT AND CHILD DEATHS.

SECTION 2. MATERNAL HISTORY

2.1.	Sa katapusang trimester sa pagmabdus nimo (sa inahan) pagpanganak, diha ba bay mga komplikasyon sama sa .	1	You (the mother) had convulsions	|___|
		2	You (the mother had high blood pressure	|___|
		3	You (the mother) had severe anemia	|___|
	**READ EACH COMPLICATION AND MARK ALL THAT APPLY.**	4	You (the mother) had diabetes	|___|
	**READ "THE MOTHER" IF THE MOTHER IS NOT THE RESPONDENT.**	5	Child delivered not head first	|___|
		6	Cord delivered first	|___|
		7	Cord around child's neck	|___|
		8	Excessive bleeding	|___|
		9	Fever during labor	|___|
		10	No complications	|___|
		11	Refuses to answer	|___|
		12	Don't know	|___|

2.2.	Unsa man ang gidugayon sa sa imong pagbuntis hangtud sa imong pagpanganak?	1	|___||___| months	
		8	Refuses to answer	|___|
		9	Don't know	|___|

IF NUMBER OF MONTHS IS KNOWN GO TO 2.4

2.3.	Sakto ba sa buwan ang imong pagpanganak, sayo ra o nasobrahan sa buwan?	1	Early	|___|
		2	On time	|___|
		3	Late	|___|
		8	Refuses to answer	|___|
		9	Don't know	|___|

2.4.	Nilihok pa ba ang bata mga pila na lang ka adlaw sa wa pa ka manganak ?	1	Yes	|___|
		2	No	|___|
		8	Refuses to answer	|___|
		9	Don't know	|___|

2.5.	Kanus-a man katapusan nimo (sa inahan) nabati-an nga nilihok ang bata? **READ "SA INAHAN" IF THE MOTHER IS NOT THE RESPONDENT.**	1	|___||___| Hours before delivery **OR**	
		2	|___||___| Days before delivery	
		8	Refuses to answer	|___|
		9	Don't know	|___|

2.6.	Kanus-a man nibuto ang panubigan – sa wala ka pa mag-bati o pagbati na nimo ? **NOTE: Labor begins when contractions are no more than 10 minutes apart.**	1	Before	|___|
		2	During	|___|
		8	Refuses to answer	|___|
		9	Don't know	|___|

IF DURING DELIVERY, GO TO 2.8

2.7.	Pila man ka adlaw sa wala pa magsugod ang imong pagbati nibuto ang imong panubigan?	1	Less than one day	|___|
		2	One day or more	|___|
		8	Refuses to answer	|___|
		9	Don't know	|___|

2.8.	Unsa man ang kolor sa nigawas aa dihang nibuto ang panubigan?	1	Green or brown	|___|
		2	Clear (normal)	|___|
		3	Others, specify ________________	|___|
		8	Refuses to answer	|___|
		9	Don't know	|___|

2.9. Baho ba ang nigawas?

1 Yes |___|
2 No |___|
8 Refuses to answer |___|
9 Don't know |___|

2.10. Unsa man kadugay ang imong pagbati usa ka nanganak ?
Less than 1 hour = 00

1 |___||___| Hours
8 Refuses to answer |___|
9 Don't know |___|

2.11. Nakadawat ka ba (ang inahan) ug bakuna sukad siya nahamtong apil na ang bakunang nadawat sa pagbuntis ?

1 Yes |___|
2 No |___|
8 Refuses to answer |___|
9 Don't know |___|

READ "ANG INAHAN' IF THE MOTHER IS NOT THE RESPONDENT.

IF"NO" OR "DON'T KNOW" OR "REFUSES TO ANSWER" GO TO QUESTION 2.13.

2.12. Pila ka doses?

1 One |___|
2 Two |___|
3 Three |___|
4 Four |___|
5 Five or more |___|
8 Refuses to answer |___|
9 Don't know |___|

2.13. Diin ka man (ang inahan) nanganak ?

1 Hospital |___|
2 Other health facility |___|
3 En route to hospital or other health facility |___|
4 Home |___|
5 Others, specify |___|
________________ |___|
8 Refuses to answer |___|
9 Don't know |___|

2.14. **FOR DELIVERIES AT HOSPITAL OR OTHER HEALTH FACILITY, RECORD HEALTH FACILITY NAME AND ADDRESS.**

2.15. Kinsa man ang nagp-anak nimo (niya) ?

1 Doctor |___|
2 Nurse/midwife |___|
3 Relative |___|
4 Self (the mother) |___|
5 Traditional birth attendant |___|
6 Others, specify |___|
____
8 Refuses to answer |___|
9 Don't know |___|

2.16. **IF NURSE/MIDWIFE IN THE COMMUNITY, RECORD HER NAME AND ADDRESS.**

2.17	.Kadtong pagpanganak ….	1	Vaginal with forceps	|___|
	**READ THE CHOICES AND MARK ONE.**	2	Vaginal without forceps	|___|
		3	Vaginal don't know	|___|
		4	C – section	|___|
		8	Refuses to answer	|___|
		9	Don't know	|___|
2.18.	Niadtong nagbati ka pa ug wala ka pa nanganak, naka-dawat ka ba ug injection ? **READ "THE MOTHER" IF THE MOTHER IS NOT THE RESPONDENT.**	1	Yes	|___|
		2	No	|___|
		8	Refuses to answer	|___|
		9	Don't know	|___|

STOP

REFER BACK TO QUESTIONS 1.15. IF YOU ANSWERED "YES" GO TO QUESTION 5.4 (SECTION 5: HEALTH RECORDS.

IF YOU ANSWERED "NO" CONTINUE TO SECTION 3: NEONATAL DEATHS.

SECTION 3. NEONATAL DEATHS

3.1.	Diha bay mga samad-samad o timailhan nga nabun-og ang bata pagkatawo ?	1	Yes	|___|
		2	No	|___|
		8	Refuses to answer	|___|
		9	Don't know	|___|
3.2.	Diha bay parte sa lawas sa bata nga dili normal adtong pagkatawo niya? (pananglitan sobra kadako, sobra ka gamay O dunay nitubo sa lawas.	1	Yes	|___|
		2	No	|___|
		8	Refuses to answer	|___|
		9	Don't know	|___|

IF "NO" OR DON'T KNOW" OR REFUSES TO ANSWER " GO TO 3.4.

|___|

3.3.	Unsa man ang di normal ?	1	Head size very small at time of birth	|___|
	**MARK ALL THE APPLY. SHOW PHOTOS.**	2	Head size very large at time of birth	|___|
		3	Mass defect on the back of the head or spine	|___|
		4	Others, specify ________________	|___|
		8	Refused to answer	|___|
3.4.	Niginhawa ba dayon ang bata pagkatawo niya ? IF NO, REFUSES TO ANSWER OR DON'T KNOW, GO TO 3.6.	1	Yes	|___|
		2	No	|___|
		8	Refuses to answer	|___|
		9	Don't know	|___|

3.5.	Naglisud ba ug ginhawa ang bata ?	1	Yes	|___|
		2	No	|___|
		8	Refuses to answer	|___|
		9	Don't know	|___|
3.6.	Diha bay gihimo aron matabangan ang bata sa paggin-hawa ?	1	Yes	|___|
		2	No	|___|
		8	Refuses to answer	|___|
		9	Don't know	|___|
3.7.	Nihilak ba dayon ang bata pag-gawas niya ? **IF "YES" GO TO 3.9.**	1	Yes	|___|
		2	No	|___|
		8	Refuses to answer	|___|
		9	Don't know	|___|
3.8.	Unsa man kadugay usa naka-hilak ang bata ? **Mark only one.** **IF "NEVER" GO TO 3.11.**	1	Within 5 mins	|___|
		2	With 6-30 mins	|___|
		3	More than 30 mins	|___|
		4	Never	|___|
		8	Refuses to answer	|___|
		9	Don't know	|___|
3.9.	Nihunong ba lang ug hilak ang bata ? **IF "NO", DON'T KNOW" OR "REFUSES TO ANSWER" GO TO 3.11.**	1	Yes	|___|
		2	No	|___|
		8	Refuses to answer	|___|
		9	Don't know	|___|
3.10.	Pila na man ka adlaw nga wala na mohilak ang bata usa siya sa namatay ?	1	Less than one day	|___|
		2	One day or more	|___|
		8	Refuses to answer	|___|
		9	Don't know	|___|
3.11.	Normal ba ang pagsuso sa bata sa unang adlaw sa iyang pagka-tawo ? **IF YES, GO TO 3.13.**	1	Yes	|___|
		2	No	|___|
		8	Refuses to answer	|___|
		9	Don't know	|___|
3.12.	Normal ba ang pagsuso sa bata? **IF "NO", DON'T KNOW" OR "REFUSES TO ANSWER" GO TO 3.17.**	1	Yes	|___|
		2	No	|___|
		8	Refuses to answer	|___|
		9	Don't know	|___|
3.13.	Diha bay panahon nga niundang ang normal nga pagsuso sa bata? **IF "NO", DON'T KNOW" OR "REFUSES TO ANSWER" GO TO 3.17.**	1	Yes	|___|
		2	No	|___|
		8	Refuses to answer	|___|
		9	Don't know	|___|
3.14.	Pila man ka adlaw human sa iyang pagkatawo, niundang ang normal nga pagsuso ? **LESS THAN 1 DAY = 00 DAYS**		**Record actual number of days**	
		1	|___||___| days	
		8	Refuses to answer	|___|
		9	Don't know	|___|
3.15.	Unsa man kadugay gikan sa pag-undang niya ug suso namatay ang bata ?	1	Less than one day	|___|
		2	One day or more	|___|
		8	Refuses to answer	|___|
		9	Don't know	|___|
3.16.	Maka-abli pa ba ang bata sa iyang baba adtong niundang na siya pagsuso ?	1	Yes	|___|
		2	No	|___|
		8	Refuses to answer	|___|
		9	Don't know	|___|

3.17.	Adtong pagkasakit sa bata padung mamatay naglisud ba ba siya ug ginhawa ?	1	Yes	|___|
		2	No	|___|
		8	Refuses to answer	|___|
		9	Don't know	|___|

IF "NO, DON'T KNOW OR REFUSES TO ANSWER" GO TO 3.20.

3.18.	Pila man ang edad sa bata nga nagsugod ang iyang lisud nga pagginhawa ? **LESS THAN 1 DAY = 00 DAYS**	1	|___||___| days	
		8	Refuses to answer	|___|
		9	Don't know	|___|
3.19.	Pila man ka adlaw nga naglisud siyag ginhawa ? **LESS THAN 1 DAY = 00 DAYS**	1	|___||___| days	
		8	Refuses to answer	|___|
		9	Don't know	|___|
3.20.	Adtong pagkasakit sa bata padung mamatay, nagpaspas ba ang iyang pagginhawa?	1	Yes	|___|
		2	No	|___|
		8	Refuses to answer	|___|
		9	Don't know	|___|

IF "NO, DON'T KNOW OR REFUSES TO ANSWER" GO TO 3.23.

3.21.	Pila man ang iyang edad nga nagsugod ang iyang paspas nga pagginhawa ? **LESS THAN 1 DAY = 00 DAYS**	1	|___||___| days	
		8	Refuses to answer	|___|
		9	Don't know	|___|
3.22.	Pila man ka adlaw nga paspas ang iyang pagginhawa ? **LESS THAN 1 DAY = 00 DAYS**	1	|___||___| days	
		8	Refuses to answer	|___|
		9	Don't know	|___|
3.23.	Adtong pagkasakit sa bata padung mamatay nag-udhak ba siya ? **SHOW PHOTO**	1	Yes	|___|
		2	No	|___|
		8	Refuses to answer	|___|
		9	Don't know	|___|
3.24.	Adtong pagkasakit sa bata padung mamatay , nag-agunto ba ang iyang pagginhawa? **DEMONSTRATE**	1	Yes	|___|
		2	No	|___|
		8	Refuses to answer	|___|
		9	Don't know	|___|
3.25.	Adtong pagkasakit sa bata padung mamatay nagkombulsyon ba kadto siya ?	1	Yes	|___|
		2	No	|___|
		8	Refuses to answer	|___|
		9	Don't know	|___|
3.26.	Adtong pagkasakit sa bata padung mamatay, gihilantan ba siya ? **IF "NO", DON'T KNOW" OR "REFUSES TO ANSWER" GO TO 3.29.**	1	Yes	|___|
		2	No	|___|
		8	Refuses to answer	|___|
		9	Don't know	|___|
3.27.	Pila man ang iyang edad pag-sugod sa hilanat ? **LESS THAN 1 DAY = 00 DAYS**	1	|___||___| days	
		8	Refuses to answer	|___|
		9	Don't know	|___|
3.28.	Pila man ka siya ka adlaw nga gihilantan ? **LESS THAN 1 DAY = 00 DAYS**	1	|___||___| days	
		8	Refuses to answer	|___|
		9	Don't know	|___|

3.29.	Adtong pagkasakit sa bata padung siyamamatay, diha bay panahon nga bugnaw na siya kung hikapon ? **IF "NO", DON'T KNOW" OR "REFUSES TO ANSWER" GO TO 3.32.**	1<br>2<br>8<br>9	Yes<br>No<br>Refuses to answer<br>Don't know	|___| |___| |___| |___|
3.30.	Pila man ang iyang edad dihang bugnaw na siya kung hikapon ? **LESS THAN 1 DAY = 00 DAYS**	1<br>8<br>9	|___||___| days<br>Refuses to answer<br>Don't know	 |___| |___|
3.31.	Pila man ka adlaw nga bugnaw Na siya kung hikapon ? **LESS THAN 1 DAY = 00 DAYS**	1<br>8<br>9	|___||___| days<br>Refuses to answer<br>Don't know	 |___| |___|
3.32.	Adtong pagkasakit sa bata padung mamatay, diha bay panahon nga luya kayo siya pagkahuman na naandan niyang kalihukan ?	1<br>2<br>8<br>9	Yes<br>No<br>Refuses to answer<br>Don't know	|___| |___| |___| |___|
3.33.	Adtong pagkasakit sa bata padung mamatay, diha bay panahon nga nawad-an siyag panimuot ug dili na mo-tubag ?	1<br>2<br>8<br>9	Yes<br>No<br>Refuses to answer<br>Don't know	|___| |___| |___| |___|
3.34.	Adtong pagkasakit sa bata padung mamatay ni burot ba ang iyang hubon? **SHOW PHOTO**	1<br>2<br>8<br>9	Yes<br>No<br>Refuses to answer<br>Don't know	|___| |___| |___| |___|
3.35.	Adtong pagkasakit sa bata padung mamatay, diha bay nana nga nigawas sa pusod ?	1<br>2<br>8<br>9	Yes<br>No<br>Refuses to answer<br>Don't know	|___| |___| |___| |___|
3.36.	Adtong pagkasakit sa bata padung mamatay, namuwa ba ang iyang pusod? **IF NO, DON'T KNOW OR REFUSES TO ANSWER GO TO 3.38.**	1<br>2<br>8<br>9	Yes<br>No<br>Refuses to answer<br>Don't know	|___| |___| |___| |___|
3.37.	Ang pagpamuwa ba sa pusod iabot na sa panit sa tiyan ?	1<br>2<br>8<br>9	Yes<br>No<br>Refuses to answer<br>Don't know	|___| |___| |___| |___|
3.38.	Adtong pagkasakit sa bata padung mamatay diha ba siyay butoy-butoy o usa ka dakong sa butoy sa panit nga dunay nana?	1<br>2<br>8<br>9	Yes<br>No<br>Refuses to answer<br>Don't know	|___| |___| |___| |___|
3.39.	Adtong pagkasakit sa bata padung mamatay diha ba siya's mga samad-samad?	1<br>2<br>8<br>9	Yes<br>No<br>Refuses to answer<br>Don't know	|___| |___| |___| |___|
3.40.	Adtong pagkasakit sa bata padung mamatay diha ba siya's parte sa panit nga namuwa ug Nagburot?	1<br>2<br>8<br>9	Yes<br>No<br>Refuses to answer<br>Don't know	|___| |___| |___| |___|

3.41. Adtong pagkasakit sa bata padung mamatay diha ba siya's parte sa panit nga nangitom ?
1 Yes |___|
2 No |___|
8 Refuses to answer |___|
9 Don't know |___|

3.42. Adtong pagkasakit sa bata padung mamatay diha ba nang-gawas nga dugo sa iyang lawas?
IF NO, DON'T KNOW OR REFUSES TO ANSWER GO TO 3.44.
1 Yes |___|
2 No |___|
8 Refuses to answer |___|
9 Don't know |___|

3.43. **RECORD FROM WHERE THE BABY BLED.**

3.44. Adtong pagkasakit sa bata padung mamatay nagkalibang na kadto siya o kaha tubig ang iyang tae?
1 Yes |___|
2 No |___|
8 Refuses to answer |___|
9 Don't know |___|

IF NO, DON'T KNOW OR REFUSES TO ANSWER GO TO 3.46.

3.45. Unsa man ang iyang pinakadag-han nga kalibang sa usa ka adlaw?
1 |___||___| stools
8 Refuses to answer |___|
9 Don't know |___|

3.46. Adtong pagkasakit sa bata padung mamatay, nagsuka ba O gisuka tanang kinaon ?
1 Yes |___|
2 No |___|
8 Refuses to answer |___|
9 Don't know |___|

3.47. Adtong pagkasakit sa bata padung mamatay, nag yellow o nag-dag ba ang iyang panit ?
1 Yes |___|
2 No |___|
8 Refuses to answer |___|
9 Don't know |___|

3.48. Adtong pagkasakit sa bata padung mamatay, nag yellow o o nag-dag ba ang iyang mata ?
1 Yes |___|
2 No |___|
8 Refuses to answer |___|
9 Don't know |___|

3.49. Himsog ba kadtong bata unya morag kalit lang nga namatay ? u
1 Yes |___|
2 No |___|
8 Refuses to answer |___|
9 Don't know |___|

STOP

END OF NEONATAL DEATHS SECTION.
GO TO SECTION 5: HEALTH RECORDS SECTION

SECTION 4. INFANT AND CHILD DEATHS

4.1. Adtong pagkasakit sa bata padung mamatay, gihilantan ba kadto siya ?
IF NO, DON'T KNOW OR REFUSES TO ANSWER GO TO 4.6.
1 Yes |___|
2 No |___|
8 Refuses to answer |___|
9 Don't know |___|

4.2. Pila ka adlaw siyang gihilantan ?
1 Less than 24 hours |___|
2 |___||___| days
8 Refuses to answer |___|
9 Don't know |___|

4.3.	Nagpadayon ba ang iyang hilanat Hangtud siya namatay ? **IF NO, DON'T KNOW OR REFUSES TO ANSWER GO TO 4.6.**	1	Yes	|___|
		2	No	|___|
		8	Refuses to answer	|___|
		9	Don't know	|___|
4.4.	Unsa man ka –grabe ang iyang Hilanat ?	1	Mild	|___|
		2	Moderate	|___|
		3	Severe	|___|
		8	Refuses to answer	|___|
		9	Don't know	|___|
4.5.	Unsa man ang klase sa iyang hilanat ?	1	Continuous	|___|
		2	On and Off	|___|
		3	Only at night	|___|
		8	Refuses to answer	|___|
		9	Don't know	|___|
4.6.	Adtong pagkasakit sa bata padung mamatay nagkalibang Na kadto siya o kaha tubig ang iyang tae? **IF NO, DON'T KNOW OR REFUSES TO ANSWER GO TO 4.12.**	1	Yes	|___|
		2	No	|___|
		8	Refuses to answer	|___|
		9	Don't know	|___|
4.7.	Unsa man ang iyang pinakadag-han nga kalibang sa usa ka adlaw?	1	|___||___| stools	
		8	Refuses to answer	|___|
		9	Don't know	|___|
4.8.	Pila man ka-adlaw siyang nagkali-bang usa siya namatay ? **IF LESS THANN 24 HRS, GO TO 4.12.**	1	Less than 24 hrs	|___|
		2	|___||___| days	
		8	Refuses to answer	|___|
		9	Don't know	|___|
4.9.	Nagpadayon ba ang iyang kali-bang hangtud siya namatay? **IF YES, GO TO 4.11.**	1	Yes	|___|
		2	No	|___|
		8	Refuses to answer	|___|
		9	Don't know	|___|
4.10.	Pila na man ka adlaw nga naundang ang iyang kalibang usa siya namatay ?stop ?	1	Less than 24 hrs	|___|
		2	|___||___| days	
		8	Refuses to answer	|___|
		9	Don't know	|___|
4.11.	Diha bay nakitang dugo sa iyang tae adtong nagkalibang siya?	1	Yes	|___|
		2	No	|___|
		8	Refuses to answer	|___|
		9	Don't know	|___|
4.12.	Adtong pagkasakit sa bata padung mamatay giubo ba siya ? **IF NO, DON'T KNOW OR REFUSES TO ANSWER GO TO 4.16.**	1	Yes	|___|
		2	No	|___|
		8	Refuses to answer	|___|
		9	Don't know	|___|
4.13.	Pila siya ka adlaw nga giubo ?	1	|___||___| days	
		8	Refuses to answer	|___|
		9	Don't know	|___|
4.14.	Grabe ba ang iyang ubo ?	1	Yes	|___|
		2	No	|___|
		8	Refuses to answer	|___|
		9	Don't know	|___|
4.15.	Nagsuka ba ang bata inig human niya ug ubo ?	1	Yes	|___|
		2	No	|___|
		8	Refuses to answer	|___|
		9	Don't know	|___|

4.16. Adtong pagkasakit sa bata padung mamatay, diha bay panahon nga naglisug siya ug ginhawa?
IF NO, REFUSES TO ANSWER OR DON'T KNOW, GO TO 4.18.

1 Yes |___|
2 No |___|
8 Refuses to answer |___|
9 Don't know |___|

4.17. Pila man ka adlaw nga morag lisud ang iyang pagginhawa?

1 |___||___| days
8 Refuses to answer |___|
9 Don't know |___|

4.18. Adtong pagkasakit sa bata padung mamatay, diha bay panahon nga paspas ang iyang pagginhawa?
IF NO, REFUSES TO ANSWER OR DON'T KNOW, GO TO 4.20.

1 Yes |___|
2 No |___|
8 Refuses to answer |___|
9 Don't know |___|

4.19. Pila man ka adlaw nga morag paspas ang iyang pagginhawa?

1 |___||___| days
8 Refuses to answer |___|
9 Don't know |___|

NOTE TO INTERVIEWER: IF BOTH 4.16. AND 4.18 ARE "NO" GO TO 4.25..

4.20. Adtong pagkasakit sa bata padung mamatay, diha bay pananon nga nag-udhak siya ?

1 Yes |___|
2 No |___|
8 Refuses to answer |___|
9 Don't know |___|

4.21. Adtong pagkasakit sa bata padung mamatay, diha bay panahon nga ang iyang pagginhawa ingon ani ug tingog :
DEMONSTRATE EACH SOUND:

4.22. Stridor

1 Yes |___|
2 No |___|
8 Refuses to answer |___|
9 Don't know |___|

4.23. Grunting

1 Yes |___|
2 No |___|
8 Refuses to answer |___|
9 Don't know |___|

4.24. Wheezing

1 Yes |___|
2 No |___|
8 Refuses to answer |___|
9 Don't know |___|

4.25. Adtong pagkasakit sa bata padung mamatay, nagkombulsyon ba kadto siya ?

1 Yes |___|
2 No |___|
8 Refuses to answer |___|
9 Don't know |___|

4.26. Nawad-an ba sa panimuot si ___ niadtong nasakit siya padung mamatay ?
IF "NO", DON'T KNOW OR REFUSES TO ANSWER, GO TO 4.28.

1 Yes |___|
2 No |___|
8 Refuses to answer |___|
9 Don't know |___|

4.27. Pila man ka oras o adlaw nga nawad-an siyang panimuot usa siya namatay ?

1 Less than 6 hours |___|
2 6 – 23 hours |___|
3 24 hours or more |___|
8 Refuses to answer |___|
9 Don't know |___|

4.28.	Adtong pagkasakit sa bata padung mamatay, nitikig o ning-gahi ba ang iyang li-og ? **DEMONSTRATE**	1	Yes	|___|
		2	No	|___|
		8	Refuses to answer	|___|
		9	Don't know	|___|
4.29.	Adtong pagkasakit sa bata padung mamatay, nibuot ba ang Iyang hubon ? **SHOW PHOTO**	1	Yes	|___|
		2	No	|___|
		8	Refuses to answer	|___|
		9	Don't know	|___|
4.30.	Adtong pagkasakit sa bata padung mamatay, diha bay Nanurok sa iyang lawas? **IF "NO", DON'T KNOW OR REFUSES TO ANSWER, GO TO 4.35.**	1	Yes	|___|
		2	No	|___|
		8	Refuses to answer	|___|
		9	Don't know	|___|
4.31.	Diin man dapit ang nanurok ?	1	Face	|___|
		2	Trunk/Abdomen	|___|
		3	Extremities	|___|
		4	Everywhere	|___|
		8	Refuses to answer	|___|
		9	Don't know	|___|
4.32.	Diin man nagsugod ang pagpa-nurok ?	1	Face	|___|
		2	Trunk/Abdomen	|___|
		3	Extremities	|___|
		4	Everywhere	|___|
		8	Refuses to answer	|___|
		9	Don't know	|___|
4.33.	Pila man ka adlaw nga duna siya Adtong nanurok usa kini nawala?	1	|___||___| days	
		8	Refuses to answer	|___|
		9	Don't know	|___|
4.34.	Diha bay sulod nga tubig-tubig Kadtong nanurok?	1	Yes	|___|
		2	No	|___|
		8	Refuses to answer	|___|
		9	Don't know	|___|
4.35.	Adtong pagkasakit sa bata padung mamatay, gagmay o niwang ba kayo ang iyang bitiis gg bukton ? **SHOW PHOTO**	1	Yes	|___|
		2	No	|___|
		8	Refuses to answer	|___|
		9	Don't know	|___|
4.36.	Adtong pagkasakit sa bata padung mamatay, nanghupon ba ang iyang bitiis ug tiil ? **IF "NO", DON'T KNOW OR REFUSES TO ANSWER, GO TO 4.38.**	1	Yes	|___|
		2	No	|___|
		8	Refuses to answer	|___|
		9	Don't know	|___|
4.37.	Pila man ka adlaw ang pagpang-hupong usa kini nawala ?	1	|___||___| days **OR**	
		2	|___||___| weeks	
		8	Refuses to answer	|___|
		9	Don't know	|___|
4.38.	Adtong pagkasakit sa bata padung mamatay, nanghup-ak ba ang iyang panit ?	1	Yes	|___|
		2	No	|___|
		8	Refuses to answer	|___|
		9	Don't know	|___|

4.39.	Nahimong puwa o yellow ba ang Kolor sa iyang buhok ?	1	Yes	|___|
		2	No	|___|
		8	Refuses to answer	|___|
		9	Don't know	|___|
4.40.	Dako ba kadtong iyang tiyan ?	1	Yes	|___|
		2	No	|___|
		8	Refuses to answer	|___|
		9	Don't know	|___|
4.41.	Adtong pagkasakit sa bata padung mamatay, luspad ba Kaayo kadto siya ?	1	Yes	|___|
		2	No	|___|
		8	Refuses to answer	|___|
		9	Don't know	|___|
4.42.	Adtong pagkasakit sa bata padung mamatay, niburot o nihupong ba ang iyang ilok ?	1	Yes	|___|
		2	No	|___|
		8	Refuses to answer	|___|
		9	Don't know	|___|
4.43.	Adtong pagkasakit sa bata padung mamatay, dihay bay Pution nga nanurok sulod sa baba o sa dila ?	1	Yes	|___|
		2	No	|___|
		8	Refuses to answer	|___|
		9	Don't know	|___|
4.44.	Adtong pagkasakit sa bata padung mamatay, dihay bay dugo nga nanggawas sa iyang lawas ?	1	Yes	|___|
		2	No	|___|
		8	Refuses to answer	|___|
		9	Don't know	|___|
	**IF "NO", DON'T KNOW OR REFUSES TO ANSWER, GO TO 4.46.**			

4.45.	**RECORD FROM WHERE S/HE BLED.**			
4.46.	Adtong pagkasakit sa bata padung mamatay, dia bay panit sa iyang lawas nga na-itum ?	1	Yes	|___|
		2	No	|___|
		8	Refuses to answer	|___|
		9	Don't know	|___|
4.47.	Nag-antos ba jkadto siya sa kadaot o accidente sama sa…	1	Road traffic crash/injury	|___|
		2	Fall	|___|
		3	Drowning	|___|
	**ASK RESPONDENT IN SEQUENCE AND MARK ALL TO WHICH THE RESPONDENT INDICATED "YES"**	4	Poisoning	|___|
		5	Bite or sting by venomous animals	|___|
		6	Fire	|___|
		7	Violence (suicide, homicide, abuse)	|___|
	**IF NO BOXES ARE MARKED, GO TO SECTION 5. IF AT LEAST ONE BOX IS MARKED CONTINUE TO 4.48.**	8	Other injury	|___|
			_______________	|___|
		9	No accident	|___|
		10	Refuses to answer	|___|
		99	Don't know	|___|
4.48.	Tinuyo ba sa laing tawo ang maong aksidente ?	1	Yes	|___|
		2	No	|___|
		8	Refuses to answer	|___|
		9	Don't know	|___|

4.49.	Pila man ka oras/adlaw nga siya nabuhi pagkahuman sa aksidente ? **LESS THAN 1 HR = 00**	1	|___||___| hours	
		2	|___||___| days	
		8	Refuses to answer	|___|
		9	Don't know	|___|

STOP

END OF INFANT AND CHILD DEATHS SECTION.
GO TO SECTION 5: HEALTH RECORDS SECTION.

SECTION 5. HEALTH RECORDS

5.1.	Gikonsulta ba kadtong sakit sa bata sa wala pa siya namatay ?	1	Yes	|___|
		2	No	|___|
		8	Refuses to answer	|___|
	**IF NO, DON'T KNOW OR REFUSES TO ANSWER, GO to 5.10.**	9	Don't know	|___|

5.2.	Diin o kang kinsa man ka kamo nikonsulta ?	1	Traditional healer	|___|
		2	Homeopath	|___|
	**CHECK ALL THAT APPLY.**	3	Religious leader	|___|
		4	Government hospital	|___|
		5	Government health center or clinic	|___|
		6	Private hospital	|___|
		7	Community based practitioner associated with health system	|___|
		8	Trained birth attendant	|___|
		9	Private physician	|___|
		10	Pharmacy, drug seller, store, market	|___|
		11	Other provider	|___|
		12	Relative, friends (outside household)	|___|
		13	Refused to answer	|___|
		99	Don't know	|___|

5.3. **RECORD THE NAME AND ADDRESS OF ANY HOSPITAL, HEALTH CENTER OR CLINIC WHERE CARE WAS SOUGHT.**

5.4.	Aduna ka bay gigunitan nga records sa center o ospital niadtong bata ?	1	Yes	|___|
		2	No	|___|
		8	Refuses to answer	|___|
	**IF NO, DON'T KNOW OR REFUSES TO ANSWER, GO TO 5.10.**	9	Don't know	|___|

5.5.	Mahimo ba akong motan-aw Iyang records?	1	Yes	|___|
		2	No	|___|
		8	Refuses to answer	|___|

IF NO, DON'T KNOW OR REFUSES TO ANSWER, GO TO 5.10. IF YES AND RESPONDENT ALLOWS YOU TO SEE THE RECORDS, TRANSCRIBE ALL THE ENTRIES.

5.6.	Record the dates of the two most recent visits..	1	|___|___|/|___|___|/|___|___|___|___| Mm dd yyyy
	**IF NOT LISTED, MARK 9999**	2	|___|___|/|___|___|/|___|___|___|___| Mm dd yyyy

5.7. Record the two most recent weights on those dates.
1 |___||___|. |___| kls
2 |___||___|. |___| kls

5.8. Record the date of the last note
1 |___|___|/|___|___|/|___|___|___|___|
Mm dd yyyy

5.9. Transcribe the note

5.10 Naisyuhan ba siya ug death certificate ?
IF NO, DON'T KNOW OR REFUSES TO ANSWER, GO TO 5.17.

1	Yes	|___|
2	No	|___|
8	Refuses to answer	|___|
9	Don't know	|___|

5.11. Mahimo ba akong motan-aw sa death certificate ?
IF NO, GO TO 5.17.

1	Yes	|___|
2	No	|___|
8	Refuses to answer	|___|

5.12. Record the immediate cause of death from the certificate.
.

5.13. Record the first underlying cause of death from the certificate

5.14. Record the second underlying cause of death from the certificate.

5.15. Record the third underlying cause of death from the certificate.

5.16. Record the contributing cause(s) of death from the certificate.

5.17. Nagpatest ka ba (ang inahan) ug HIV sukad ?
IF NO, DON'T KNOW OR REFUSES TO ANSWER GO TO 5.19.

1	Yes	|___|
2	No	|___|
8	Refuses to answer	|___|
9	Don't know	|___|

5.18. Positive ba ang results sa test?

1	Yes	|___|
2	No	|___|
8	Refuses to answer	|___|
9	Don't know	|___|

5.19. Nasultihan ka na ba sukad (ang inahan) sa health worker nga duna kay (siya's) HIV /AIDS?

1	Yes	|___|
2	No	|___|
8	Refuses to answer	|___|
9	Don't know	|___|

SECTION 6. OPEN ENDED RESPONSE AND INTERVIEWERS COMMENTS AND OBSERVATIONS

INTRUCTIONS TO INTERVIEWERS: Ask the respondent: Thank you for the patient responses to this exhaustive set of questions. Could you please summarize or tell us in your own words, any additional information about the illness and/or death of your loved one?

TO THE INTERVIEWER: Write down what the respondent tells you in his/her own words. Do not prompt except for asking whether there was anything else after the respondent finishes. While recording, underline any unfamiliar terms. You may also use this space to write down your comments and observations about the interview.

END OF INTERVIEW
THANK THE RESPONDENT FOR PARTICIPATION.

SECTION 6. OPEN ENDED RESPONSE AND INTERVIEWERS COMMENTS AND OBSERVATIONS

INTRUCTIONS TO INTERVIEWERS: Translate into English what you have recorded in the preceding page.

POPULATION HEALTH METRICS RESEARCH CONSORTIUM
ADULT AND ADOLESCENT VERBAL AUTOPSY MODULE

SECTION 1. HISTORY OF CHRONIC CONDITIONS OF THE DECEASED

1.1. Duna ba sa bisan unsa sa mosunod ang namatay ?

Hubak	1	Yes	|___|
	2	No	|___|
	8	Refuses to answer	|___|
	9	Don't know	|___|
Arthritis	1	Yes	|___|
	2	No	|___|
	8	Refuses to answer	|___|
	9	Don't know	|___|
Cancer	1	Yes	|___|
	2	No	|___|
	8	Refuses to answer	|___|
	9	Don't know	|___|
TB o Tibihon	1	Yes	|___|
	2	No	|___|
	8	Refuses to answer	|___|
	9	Don't know	|___|
Malimtanon	1	Yes	|___|
	2	No	|___|
	8	Refuses to answer	|___|
	9	Don't know	|___|
Magul-anon/Masulob-on	1	Yes	|___|
	2	No	|___|
	8	Refuses to answer	|___|
	9	Don't know	|___|
Diabetes	1	Yes	|___|
	2	No	|___|
	8	Refuses to answer	|___|
	9	Don't know	|___|
Patolon	1	Yes	|___|
	2	No	|___|
	8	Refuses to answer	|___|
	9	Don't know	|___|
Sakit sa kasingkasing	1	Yes	|___|
	2	No	|___|
	8	Refuses to answer	|___|
	9	Don't know	|___|
Taas ang blood pressure	1	Yes	|___|
	2	No	|___|
	8	Refuses to answer	|___|
	9	Don't know	|___|
Sobra ang pagkatambok	1	Yes	|___|
	2	No	|___|
	8	Refuses to answer	|___|
	9	Don't know	|___|
Stroke	1	Yes	|___|
Atake	2	No	|___|
	8	Refuses to answer	|___|
	9	Don't know	|___|
Chronic obstructive pulmonary	1	Yes	|___|
Disease (COPD)	2	No	|___|
	8	Refuses to answer	|___|
	9	Don't know	|___|
AIDS	1	Yes	|___|
	2	No	|___|
	8	Refuses to answer	|___|
	9	Don't know	|___|

SECTION 2. SYMPTOM CHECKLIKST

2.1.	Unsa man kadugay nga nasakit si ____ usa siya namatay ?	1	|___||___| months	
		2	|___||___| days	
		8	Refuses to answer	|___|
		9	Don't know	|___|
2.2.	Gihilantan ba kadto siya ? **IF NO, REFUSES TO ANSWER OR DON'T KNOW GO TO 2.7.**	1	Yes	|___|
		2	No	|___|
		8	Refuses to answer	|___|
		9	Don't know	|___|
2.3.	Pila man siya ka adlaw nga gihilantan ?	1	|___||___| days	
		8	Refuses to answer	|___|
		9	Don't know	|___|
2.4.	Unsa man ka grabe ang iyang hilanat ?	1	Mold	|___|
		2	Moderate	|___|
		3	Severe	|___|
		8	Refuses to answer	|___|
		9	Don't know	|___|
2.5.	Unsa man ang klase sa iyang hilanat ?	1	Continuous	|___|
		2	On and off	|___|
		3	Only at night	|___|
		8	Refuses to answer	|___|
		9	Don't know	|___|
2.6.	Giubanan ba ug pagpaningot ang iyang hilanat ?	1	Yes	|___|
		2	No	|___|
		8	Refuses to answer	|___|
		9	Don't know	|___|
2.7.	Diha bay nanurok adtong namatay ? **IF NO, REFUSES TO ANSWER OR DON'T KNOW GO TO 2.10.**	1	Yes	|___|
		2	No	|___|
		8	Refuses to answer	|___|
		9	Don't know	|___|
2.8.	Pila man ka adlaw kadtong iyang mga panurok ?	1	|___||___| days	1
		8	Refuses to answer	8
		9	Don't know	9
2.9.	Diin man dapit ang iyang mga panurok ?	1	Face	|___|
		2	Trunk	|___|
		3	Extremities	|___|
		4	Everywhere	|___|
		5	Others, specify ______________	|___|
		8	Refuses to answer	|___|
		9	Don't know	|___|
2.10.	Diha ba siyay mga botoy-botoy sa iyang panit ? **IF NO, REFUSES TO ANSWER OR DON'T KNOW GO TO 2.12.**	1	Yes	|___|
		2	No	|___|
		8	Refuses to answer	|___|
		9	Don't know	|___|
2.11.	Diha bay sulod nga nana o tubig kadtong iyang butoy-butoy ?	1	Yes	|___|
		2	No	|___|
		8	Refuses to answer	|___|
		9	Don't know	|___|
2.12.	Diha ba siyay pangatol sa panit?	1	Yes	|___|
		2	No	|___|
		8	Refuses to answer	|___|
		9	Don't know	|___|
2.13.	Diha ba siyay ulcer o dakong samad sa tiil ? **IF NO, REFUSES TO ANSWER OR DON'T KNOW GO TO 2.16.**	1	Yes	|___|
		2	No	|___|
		8	Refuses to answer	|___|
		9	Don't know	|___|
2.14.	Nagnana ba kadtong iyang samad ? **IF NO, REFUSES TO ANSWER OR DON'T KNOW GO TO 2.16.**	1	Yes	|___|
		2	No	|___|
		8	Refuses to answer	|___|
		9	Don't know	|___|
2.15.	Pila man ka adlaw nga nagnana ang iyang samad?	1	|___||___| days	
		8	Refuses to answer	|___|
		9	Don't know	|___|

2.16.	Nakasinati ba siya ug pagpamin-hod sa iyang tiil ?	1	Yes	|___|
		2	No	|___|
		8	Refuses to answer	|___|
		9	Don't know	|___|
2.17.	Nanglagom ba kadtong iyang Ngabil ?	1	Yes	|___|
		2	No	|___|
		8	Refuses to answer	|___|
		9	Don't know	|___|
2.18.	Niubos ba kaayo ang iyang timbang sa miaging 3 ka bulan? **IF NO, REFUSES TO ANSWER OR DON'T KNOW GO TO 2.20.**	1	Yes	|___|
		2	No	|___|
		8	Refuses to answer	|___|
		9	Don't know	|___|
2.19.	Unsa man kadako ang pag-ubos sa iyang timbang ?	1	Slight	|___|
		2	Moderate	|___|
		3	Large	|___|
		8	Refuses to answer	|___|
		9	Don't know	|___|
2.20.	Luspad ba siya tan-awon ?	1	Yes	|___|
		2	No	|___|
		8	Refuses to answer	|___|
		9	Don't know	|___|
2.21.	Nag-yellow ba ang iyang mata? **IF NO, REFUSES TO ANSWER OR DON'T KNOW GO TO 2.23.**	1	Yes	|___|
		2	No	|___|
		8	Refuses to answer	|___|
		9	Don't know	|___|
2.22.	Unsa mankadugay nga nag-yellow iyang mata usa siya namatay ?	1	|___||___| Months	
		2	|___||___| days	
		8	Refuses to answer	|___|
		9	Don't know	|___|
2.23.	Nanghupong ba kadtong iyang tikod ? **IF NO, REFUSES TO ANSWER OR DON'T KNOW GO TO 2.25.**	1	Yes	|___|
		2	No	|___|
		8	Refuses to answer	|___|
		9	Don't know	|___|
2.24.	Unsa man sab kadugay nga nanghupong ang iyang tikod usa siya namatay ?	1	|___||___| Months	
		2	|___||___| days	
		8	Refuses to answer	|___|
		9	Don't know	|___|
2.25.	Nanghupong ba sab ang iyang nawong ? **IF NO, REFUSES TO ANSWER OR DON'T KNOW GO TO 2.27.**	1	Yes	|___|
		2	No	|___|
		8	Refuses to answer	|___|
		9	Don't know	|___|
2.26.	Unsa man sab kadugay nga nanghupong ang iyang nawong usa siya namatay ?	1	|___||___| Months	
		2	|___||___| days	
		8	Refuses to answer	|___|
		9	Don't know	|___|
2.27.	Nanghupong ba ang iyang tibuok lawas ? **IF NO, REFUSES TO ANSWER OR DON'T KNOW GO TO 2.29.**	1	Yes	|___|
		2	No	|___|
		8	Refuses to answer	|___|
		9	Don't know	|___|
2.28.	Unsa man sab kadugay nga nanghupong ang iyang lawas usa siya namatay ?	1	|___||___| Months	
		2	|___||___| days	
		8	Refuses to answer	|___|
		9	Don't know	|___|
2.29.	Diha ba lusay ang iyang li-og ?	1	Yes	|___|
		2	No	|___|
		8	Refuses to answer	|___|
		9	Don't know	|___|
2.30.	Diha ba lusay ang iyang ilok ?	1	Yes	|___|
		2	No	|___|
		8	Refuses to answer	|___|
		9	Don't know	|___|
2.31.	Diha ba lusay ang iyang bugan ?	1	Yes	|___|
		2	No	|___|
		8	Refuses to answer	|___|
		9	Don't know	|___|

2.32.	Giubo ba kadto siya ?<br>**IF NO, REFUSES TO ANSWER OR DON'T KNOW GO TO 2.36.**	1	Yes	|___|
		2	No	|___|
		8	Refuses to answer	|___|
		9	Don't know	|___|
2.33.	Unsa man kadugay ang iyang ubo usa siya namatay ?	1	|___||___| Months	
		2	|___||___| days	
		8	Refuses to answer	|___|
		9	Don't know	|___|
2.34.	Diubanan bag plema ang iyang ubo ?	1	Yes	|___|
		2	No	|___|
		8	Refuses to answer	|___|
		9	Don't know	|___|
2.35.	Nag-ubo ba siyag dugo ?	1	Yes	|___|
		2	No	|___|
		8	Refuses to answer	|___|
		9	Don't know	|___|
2.36.	Nagpakita ba siyag lisud nga pagginhawa ?	1	Yes	|___|
		2	No	|___|
	**IF NO, REFUSES TO ANSWER OR DON'T KNOW GO TO 2.40.**	8	Refuses to answer	|___|
		9	Don't know	|___|
2.37.	Unsa man kadugay ang iyang lisud nga pagginhawa usa siya namatay ?	1	|___||___| Months	
		2	|___||___| days	
		8	Refuses to answer	|___|
		9	Don't know	|___|
2.38.	Nagundang-undang ba 0 derecho ang ang iyang paglisud ug ginhawa?	1	Continuous	|___|
		2	On and Off	|___|
		8	Refuses to answer	|___|
		9	Don't know	|___|
2.39.	Unsa mang posisyona morag mograbe ang kalisud sa iyang pagginhawa?<br>**READ EACH CHOICE IN SEQUENCE.**	1	Lying	|___|
		2	Sitting	|___|
		3	Walking/during Exertion	|___|
		4	Didn't matter	|___|
		8	Refuses to answer	|___|
		9	Don't know	|___|
2.40.	Paspas ba kadtong iyang pagginhawa?	1	Yes	|___|
		2	No	|___|
		8	Refuses to answer	|___|
		9	Don't know	|___|
2.41.	Unsa man kadugay ang iyang paspas nga pagginhawa usa siya namatay ?	1	|___||___| Months	
		2	|___||___| days	
		8	Refuses to answer	|___|
		9	Don't know	|___|
2.42.	Mora bag managhoy siya kung sa iyang pagginhawa?<br>**DEMONSTRATE**	1	Yes	|___|
		2	No	|___|
		8	Refuses to answer	|___|
		9	Don't know	|___|
2.43.	Nakasinati ba siyag pagsakit sa iyang dughan mga usa ka buwan sa wala pa siya namatay?<br>**IF NO, REFUSES TO ANSWER OR DON'T KNOW GO TO 2.47.**	1	Yes	|___|
		2	No	|___|
		8	Refuses to answer	|___|
		9	Don't know	|___|
2.44.	Unsa man kadugay kadtong sakit sa dughan usa siya namatay?	1	Less than 30 mins	|___|
		2	30 mins to 24 hrs	|___|
		3	More than 24 hrs	|___|
		8	Refuses to answer	|___|
		9	Don't know	|___|
2.45.	Kadtong pagsakit sa dughan nahitabo ba kung duna siyay gibuhat nga bug-at ?	1	Yes	|___|
		2	No	|___|
		8	Refuses to answer	|___|
		9	Don't know	|___|

2.46.	Diin man dapit ang sakit ?<br><br>**READ EACH CHOICE IN SEQUENCE.**	1	Upper or middle Chest	|___|
		2	Lower chest	|___|
		3	Left arm	|___|
		4	Others specify ________________	|___|
		8	Refuses to answer	|___|
		9	Don't know	|___|
2.47.	Nagkalibang ba kadto siya o kaha tubigon ang iyang tae?<br>**IF NO, REFUSES TO ANSWER OR DON'T KNOW GO TO 2.49.**	1	Yes	|___|
		2	No	|___|
		8	Refuses to answer	|___|
		9	Don't know	|___|
2.48.	Unsa man kadugay nga nagkalibang siya o tubigon ang iyang tae usa siya namatay ?	1	|___||___| days	|___|
		8	Refuses to answer	|___|
		9	Don't know	|___|
2.49.	Diha bay nausab sa iyang naandang batasan o schedule sa pagpangasilyas?	1	Yes	|___|
		2	No	|___|
		8	Refuses to answer	|___|
		9	Don't know	|___|
2.50.	Diha bay dugo sa iyang tae ?<br>**IF NO, REFUSES TO ANSWER OR DON'T KNOW GO TO 2.52.**	1	Yes	|___|
		2	No	|___|
		8	Refuses to answer	|___|
		9	Don't know	|___|
2.51.	Diha bay dugo sa iyang tae hangtud sa iyang pagkamatay?	1	Yes	|___|
		2	No	|___|
		8	Refuses to answer	|___|
		9	Don't know	|___|
2.52.	Niundang o dili na ba siya maka-pangihi ?	1	Yes	|___|
		2	No	|___|
		8	Refuses to answer	|___|
		9	Don't know	|___|
2.53.	Nagsuka ba kadto siya usa ka semana sa wala pa siya mamatay?<br>**IF NO, REFUSES TO ANSWER OR DON'T KNOW GO TO 2.57.**	1	Yes	|___|
		2	No	|___|
		8	Refuses to answer	|___|
		9	Don't know	|___|
2.54.	Unsa kadugay ang iyang pagsuka usa siya namatay?	1	|___||___| days	
		2	|___||___| hours	
		8	Refuses to answer	|___|
		9	Don't know	|___|
2.55.	Diha bay niuban nga dugo sa Iyang suka ?	1	Yes	|___|
		2	No	|___|
		8	Refuses to answer	|___|
		9	Don't know	|___|
2.56.	Itum ba ang kolor sa iyang suka ?	1	Yes	|___|
		2	No	|___|
		8	Refuses to answer	|___|
		9	Don't know	|___|
2.57.	Naglisud ba siya ug tulon ug bisan unsa ?<br>**IF NO, REFUSES TO ANSWER OR DON'T KNOW GO TO 2.60.**	1	Yes	|___|
		2	No	|___|
		8	Refuses to answer	|___|
		9	Don't know	|___|
2.58.	Unsa man kadugay nga naglisud siya ug tulon usa siya namatay?	1	|___||___| months	
		2	|___||___| days	
		8	Refuses to answer	|___|
		9	Don't know	|___|
2.59.	Ang lisud ba niya sa pagtulon sa pagkaon lang, tubig o sa pagkaon UG tubig ?	1	Solids	
		2	Fluids	
		3	Both	
		8	Refuses to answer	|___|
		9	Don't know	|___|

2.60.	Nakasinati ba sab siyag sakit kung motulon siya ?	1	Yes	|___|
		2	No	|___|
		8	Refuses to answer	|___|
		9	Don't know	|___|
2.61.	Nagsakit ba kadtong iyang tiyan? **IF NO, REFUSES TO ANSWER OR DON'T KNOW GO TO 2.64.**	1	Yes	|___|
		2	No	|___|
		8	Refuses to answer	|___|
		9	Don't know	|___|
2.62.	Unsa man kadugay ang pagsakit sa iyang tiyan usa siya namatay ?	1	|___||___| days	
		2	|___||___| hours	
		8	Refuses to answer	|___|
		9	Don't know	|___|
2.63.	Ang pagasakit bas a iyang tiyan sa dapit sa ibabaw o ubos nga parte sa tiyan?	1	Upper belly	|___|
		2	Lower belly	|___|
		8	Refuses to answer	|___|
		9	Don't know	|___|
2.64.	Dako ba kaayo ang iyang tiyan kay sa kasagaran ? **IF NO, REFUSES TO ANSWER OR DON'T KNOW GO TO 2.67.**	1	Yes	|___|
		2	No	|___|
		8	Refuses to answer	|___|
		9	Don't know	|___|
2.65.	Unsa man kadugay nga nidako ang iyang tiyan usa siya namatay ?	1	|___||___| months	
		2	|___||___| days	
		8	Refuses to answer	|___|
		9	Don't know	|___|
2.66.	Unsa man ka paspas o kadali ang pagdako sa iyang tiyan ?	1	Rapidly	|___|
		2	Slowly	|___|
		8	Refuses to answer	|___|
		9	Don't know	|___|
2..67.	Diha bay bugon sa iyang tiyan ? **IF NO, REFUSES TO ANSWER OR DON'T KNOW GO TO 2.69.**	1	Yes	|___|
		2	No	|___|
		8	Refuses to answer	|___|
		9	Don't know	|___|
2.68.	Unsa naman kadugay nga dunay bugon sa iyang tiyan usa siya namatay ?	1	|___||___| months	
		2	|___||___| days	
		8	Refuses to answer	|___|
		9	Don't know	|___|
2.69.	Nagsakit ba kadtong iyang ulo ? **IF NO, REFUSES TO ANSWER OR DON'T KNOW GO TO 2.72.**	1	Yes	|___|
		2	No	|___|
		8	Refuses to answer	|___|
		9	Don't know	|___|
2.70.	Unsa man kadugay nga nagasa-kit ang iyang ulo usa siya namatay ?	1	|___||___| hours	
		2	|___||___| days	
		8	Refuses to answer	|___|
		9	Don't know	|___|
2.71.	Kadtong pagsugod sa sakit sa ulo niya kalit o inanay ?	1	Fast	|___|
		2	Slow	|___|
		8	Refuses to answer	|___|
		9	Don't know	|___|
2.72.	Nagtikig o nigahi ba kadtong Iyang liog ? **IF NO, REFUSES TO ANSWER OR DON'T KNOW GO TO 2.74.**	1	Yes	|___|
		2	No	|___|
		8	Refuses to answer	|___|
		9	Don't know	|___|
2.73.	Unsa man kadugay nga nagtikig ang iyang liog usa siya namatay?	1	|___||___| months	
		2	|___||___| days	
		8	Refuses to answer	|___|
		9	Don't know	|___|
2.74.	Diha bay panahon nga nawad-an siyag panimuot? **IF NO, REFUSES TO ANSWER OR DON'T KNOW GO TO 2.78.**	1	Yes	|___|
		2	No	|___|
		8	Refuses to answer	|___|
		9	Don't know	|___|
2.75.	Kadtong pagkawa sa iyang panimuot kalit o hinay-hinay ang Pagsugod?	1	Suddenly	|___|
		2	Slowly	|___|
		8	Refuses to answer	|___|
		9	Don't know	|___|

2.76.	Unsa man kadugay nga nawad-an siyang panimuot usa siya namatay ?	1	|___||___| hours	
		2	|___||___| days	
		8	Refuses to answer	|___|
		9	Don't know	|___|
2.77.	Nagpadayon ba kadto hangtud siya namatay ?	1	Yes	|___|
		2	No	|___|
		8	Refuses to answer	|___|
		9	Don't know	|___|
2.78.	Niadtong mga 3 ka buwan sa pa siya mamatay, nakasinati ba si ____ ug panahon nga mora siyag nalibog?<br>**IF NO, REFUSES TO ANSWER OR DON'T KNOW GO TO 2.81.**	1	Yes	|___|
		2	No	|___|
		8	Refuses to answer	|___|
		9	Don't know	|___|
2.79.	Unsa man kadugay nga mora siyag nalibog usa siya namatay?	1	|___||___| hours	
		2	|___||___| days	
		3	|___||___| months	
		8	Refuses to answer	|___|
		9	Don't know	|___|
2.80.	Kadtong pagkalibog niya, kalit O hinay-hinay ang pagsugod Adto ?	1	Suddenly	|___|
		2	Slowly	|___|
		8	Refuses to answer	|___|
		9	Don't know	|___|
2.81.	Niadtong mga 3 ka buwan sa pa mamatay si ____, nahimo ba siyang malimtanon ?	1	Yes	|___|
		2	No	|___|
		8	Refuses to answer	|___|
		9	Don't know	|___|
2.82.	Nagkombulsyon ba kadto siya?<br>**IF NO, REFUSES TO ANSWER OR DON'T KNOW GO TO 2.85.**	1	Yes	|___|
		2	No	|___|
		8	Refuses to answer	|___|
		9	Don't know	|___|
2.83.	Unsa man ang gidugayon nga nagkombulsyon siya sap w siya mamatay ?	1	|___||___| mins	
		2	|___||___| hours	
		8	Refuses to answer	|___|
		9	Don't know	|___|
2.84.	Nwad-an ba siya ug panimuot pagkahuman gyud sa iyang kombulsyon ?	1	Yes	|___|
		2	No	|___|
		8	Refuses to answer	|___|
		9	Don't know	|___|
2.85.	Na-paralyze ba kakdto si ____?<br>**IF NO, REFUSES TO ANSWER OR DON'T KNOW GO TO 2.88.**	1	Yes	|___|
		2	No	|___|
		8	Refuses to answer	|___|
		9	Don't know	|___|
2.86.	Unsa man ang gidugayon sa iyang pagkaparalyze usa siya namatay?	1	|___||___| days	
		2	|___||___| months	
		3	|___||___| years	
		8	Refuses to answer	|___|
		9	Don't know	|___|
2.87.	Unsa man ang mga parte nga na-paralyze?<br>**READ THROUGH THE LIST IN SEQUENCE AND MARK ALL THAT APPLY.**	1	Right side (hand and leg)	|___|
		2	Left side)hand and leg)	|___|
		3	Lower part of the body	|___|
		4	Upper part of the body	|___|
		5	One leg only	|___|
		6	One arm only	|___|
		7	Whole body	|___|
		8	Refuses to answer	|___|
		9	Don't know	|___|

2.88. **STOP**

IF THE RESPONDENT IS FEMALE, THEN CONTINUE TO SECTION 3. QUESTIONS FOR WOMEN.

IF THE RESPONDENT IS MALE, GO TO SECTION 4.: ALCOHOL AND TOBACCO

SECTION 3. QUESTIONS FOR WOMEN

3.1.	Nihubag ba o duna bay bugon ang suso ni _____?	1	Yes	|___|
		2	No	|___|
		8	Refuses to answer	|___|
		9	Don't know	|___|
3.2.	Ang iyang suso mora bag dunay samad-samad morag panit sa kahel ?	1	Yes	|___|
		2	No	|___|
		8	Refuses to answer	|___|
		9	Don't know	|___|
3.3.	Natural ba ang pagka-undang sa regla ni ___ tungod kay nag-menopause na siya ? **IF NO, REFUSES TO ANSWER OR DON'T KNOW, GO TO 3.5.**	1	Yes	|___|
		2	No	|___|
		8	Refuses to answer	|___|
		9	Don't know	|___|
3.4.	Diha bay panahon nga dihay dugo nga nigawas sa pwerta ni ___ bisan wan a siya mag-regla ? **IF NO, REFUSES TO ANSWER OR DON'T KNOW, GO TO SECTION 4.**	1	Yes	|___|
		2	No	|___|
		8	Refuses to answer	|___|
		9	Don't know	|___|
3.5.	Kadto si ___ diha bay kasinatian nga dunay dugo mogawas sa iyang pwerta maski wala siya reglaha ?	1	Yes	|___|
		2	No	|___|
		8	Refuses to answer	|___|
		9	Don't know	|___|
3.6.	Diha bay kusog kaayo nga dugo nga nigawas sa iyang pwerta usa ka semana sa wala pa siya mamatay ?	1	Yes	|___|
		2	No	|___|
		8	Refuses to answer	|___|
		9	Don't know	|___|
3.7.	Pagkamatay ni ____, wala ba siya tungahi sa iyang regla? **IF NO, REFUSES TO ANSWER OR DON'T KNOW, GO TO 3.10.**	1	Yes	|___|
		2	No	|___|
		8	Refuses to answer	|___|
		9	Don't know	|___|
3.8.	Pila na man ka semana nga wala siya tungahi ?	1	|___||___| weeks	
		8	Refuses to answer	|___|
		9	Don't know	|___|
3.9.	Diha bay grabe kaayong sakit sa iyang tiyan niadtong padung na siyang mamatay ?	1	Yes	|___|
		2	No	|___|
		8	Refuses to answer	|___|
		9	Don't know	|___|
3.10.	Buntis o mabdus pa kaha kadto siya pagkamatay ? **IF NO, REFUSES TO ANSWER OR DON'T KNOW, GO TO 3.17.**	1	Yes	|___|
		2	No	|___|
		8	Refuses to answer	|___|
		9	Don't know	|___|
3.11	Pila man siya ka buwan nga buntis?		|___| months	
3.12	Nag- bleeding o spotting ba siya adtong buntis pa siya ?	1	Yes	|___|
		2	No	|___|
		8	Refused to answer	|___|
		9	Don't know	|___|
3.13	Namatay ba siya adtong nakuhaan siya? **IF NO, REFUSED TO ANSWER OR DON'T KNOW GO TO QUES 3.15.**	1	Yes	|___|
		2	No	|___|
		8	Refused to answer	|___|
		9	Don't know	|___|
3.14	Sobra o grabe ba ang iyang pagdugo o pag haemorrhage adtong nakuhaan siya ?	1	Yes	|___|
		2	No	|___|
		8	Refused to answer	|___|
		9	Don't know	|___|
	**GO TO SECTION 4**			
3.15	Namatay ba siya human siya nakuhaan ? **IF NO, REFUSED TO ANSWER OR DON'T KNOW GO TO QUES 3.17.**	1	Yes	|___|
		2	No	|___|
		8	Refused to answer	|___|
		9	Don't know	|___|

3.16	Sobra o grabe ba ang pagdugo o pag haemorrhage niya pagka-Human siya nakuhaai?	1	Yes	|___|
		2	No	|___|
		8	Refused to answer	|___|
		9	Don't know	|___|

GO TO SECTION 4

3.17	Namatay ba siya sulod sa 6 ka semana human siya nakuhaan?	1	Yes	|___|
		2	No	|___|
		8	Refused to answer	|___|
		9	Don't know	|___|

IF NO, REFUSED TO ANSWER OR DON'T KNOW GO TO QUES 3.19.

3.18	Baho ba ang nigawas sa iyang pwerta sulod sa 6 ka semana human siya nakuhaan ?	1	Yes	|___|
		2	No	|___|
		8	Refused to answer	|___|
		9	Don't know	|___|

GO TO SECTION 4

3.19	Namatay ba siya samtang siya nagbati? *(Labor is the period of time by which contraction are less than 10 minutes* apart)	1	Yes	|___|
		2	No	|___|
		8	Refused to answer	|___|
		9	Don't know	|___|

IF NO, REFUSED TO ANSWER OR DON'T KNOW GO TO QUES 3.22.

3.20 Pila siya ka oras nagbati? |___||___| hours

3.21	Sobra o grabe ba ang pagdugo pag haemorrhage niadtong Nagbati pa siya ?	1	Yes	|___|
		2	No	|___|
		8	Refused to answer	|___|
		9	Don't know	|___|

GO TO SECTION 4

3.22	Namatay ba siya pagoanganak gyud niya ?	1	Yes	|___|
		2	No	|___|
		8	Refused to answer	|___|
		9	Don't know	|___|
				|___|

IF NO, REFUSED TO ANSWER OR DON'T KNOW GO TO QUES 3.24.

3.23	Sobra o grabe ba ang pagdugo pag haemorrhage niadtong nanganak siya ?	1	Yes	|___|
		2	No	|___|
		8	Refused to answer	|___|
		9	Don't know	|___|

GO TO SECTION 4

3.24	Namatay ba siya pagkahuman niyag panganak?	1	Yes	|___|
		2	No	|___|
		8	Refused to answer	|___|
		9	Don't know	|___|

IF NO, REFUSED TO ANSWER OR DON'T KNOW GO TO QUES 3.26

3.25	Sobra o grabe ba ang pagdugo o pag haemorrhage niadtong Pagkahuman niyag panganak?	1	Yes	|___|
		2	No	|___|
		8	Refused to answer	|___|
		9	Don't know	|___|

GO TO SECTION 4

3.26	Namatay ba siya sulod sa 6 ka semana pagkahuman niyag panganak?	1	Yes	|___|
		2	No	|___|
		8	Refused to answer	|___|
		9	Don't know	|___|

IF NO, REFUSED TO ANSWER OR DON'T KNOW GO TO SECTION 4

3.27	Baho ba ang nigawas sa iyang pwerta sulod sa 6 ka semana human niyag panganak ?	1	Yes	|___|
		2	No	|___|
		8	Refused to answer	|___|
		9	Don't know	|___|

GO TO SECTION 4

SECTION 4. ALCOHOL AND TOBACCO

4.1.	Nanigarilyo o nagtabako ba kadto si _____ ?<br>**IF NO, REFUSES TO ANSWER OR DON'T KNOW, GO TO 4.5.**	1<br>2<br>8<br>9	Yes<br>No<br>Refuses to answer<br>Don't know	|___| |___| |___| |___|
4.2.	Unsa man nga klase ang iyang gipanigarilyo o gitababako ?<br><br>**IF YES TO CIGARETTES GO TO QUES 4.4.**<br><br>**IF YES TO PIPE OR CHEWING TOBACCO, CONTINUE.**<br><br>**IF OTHERS, REFUSES TO ANSWER OR DON'T KNOW GO TO**	1<br>2<br>3<br>4<br><br>5<br><br>8<br>9	Cigarettes<br>Pipe<br>Chewing tobacco<br>Local form of<br>Tobacco<br>Others, specify<br>________________<br>Refuses to answer<br>Don't know	|___| |___| |___| |___| |___| |___| <br>|___| |___|
4.3.	Kapila man siya mag 'mama' ug tabako o magtabako ginamit ang iyang pipe sa usa ka adlaw?	1<br><br>8<br>9	|___||___| chews or dips a day<br>Refuses to answer<br>Don't know	 <br>|___| |___|
4.4.	Pila man ka stick sa sigarilyo ang iyang mahurot sa usang adlaw?<br>**IF NO, REFUSES TO ANSWER OR DON'T KNOW GO TO SECTION 5.**	1<br>8<br>9	|___||___| sticks<br>Refuses to answer<br>Don't know	 |___| |___|
4.5.	Moinom ba ug alak kadto si _____ ?<br><br>**IF NO, REFUSES TO ANSWER OR DON'T KNOW GO TO SECTION 5.**	1<br>2<br>3<br>4	Yes<br>No<br>Refuses to answer<br>Don't know	|___| |___| |___| |___|
4.6.	Unsa man ang imong bana-bana sa gidaghanon sa alak nga Iyang mahurot sa usa ka adlaw ?	1<br>2<br>3<br>8<br>9	Low<br>Moderate<br>High<br>Refuses to answer<br>Don't know	|___| |___| |___| |___| |___|

SECTION 5. INJURIES/ACCIDENTS

5.1.	Kadto si ___ naaksidente ba sama sa …<br><br>**ASK RESPONDENT EACH IN SEQUENCE AND MARK ALL TO WHICH THE RESPONDENT INDICATED YES.**<br><br>**IF NO BOXES ARE CHECKED GO TO SECTION 6. IF AT LEAST ONE BOX IS CHECKED, CONTINUE TO 5.2.**	1<br>2<br>3<br>4<br>5<br><br>6<br>7<br><br>8<br>9	Road traffic injury<br>Fall<br>Drowning<br>Poisoning<br>Bite or sting by venomous animal<br>Burns<br>Violence (suicide, homicide, abuse)<br>NO<br>Other injuries, specify<br>________________	|___| |___| |___| |___| |___| <br>|___| |___| <br>|___| |___|
5.2.	Kadtong aksidente gihimo ba sa namatay sa iyang kaugalingon?	1<br>2<br>8<br>9	Yes<br>No<br>Refuses to answer<br>Don't know	|___| |___| |___| |___|
5.3.	Kadtong aksidente tinuyo bang gihimo sa laing tawo ?	1<br>2<br>8<br>9	Yes<br>No<br>Refuses to answer<br>Don't know	|___| |___| |___| |___|
5.4.	Unsa man ka kadugay siyang nabuhi human sa iyang aksidente?	1<br>2<br>3<br>4<br>8<br>9	|___||___| hours<br>|___||___| days<br>|___||___| months<br>|___||___| years<br>Refuses to answer<br>Don't know	 <br><br><br>|___| |___|

SECTION 6. HEALTH RECORDS

6.1. Gikonsulta ba kadtong sakit ni ____ sa gawas sa balay?

IF NO, REFUSES TO ANSWER OR DON'T KNOW, GO TO SECTION 7.

1	Yes	|___|
2	No	|___|
8	Refuses to answer	|___|
9	Don't know	|___|
.		

6.2. Diin o kang kinsa man siya gikonsulta ?

1	Traditional healer	|___|
2	Homeopath	|___|
3	Religious leader	|___|
4	Govt hospital	|___|
5	Govt clinic or health center	|___|
6	Private hospital	|___|
7	Community based Practitioners asso-ciated with the health system	|___|
8	Trained birth Attendant	|___|
9	Private physician	|___|
10	Pharmacy, drug seller, store, market	|___|
11	Other provider ________________	|___|
12	Relatives, friends	|___|
13	Refuses to answer	|___|
99	Don't know	

6.3. **RECORD THE NAME AND ADDRESS OF ANY HOSPITAL, HEALTH CENTER OR CLINIC WHERE CARE WAS SOUGHT.**

6.4. Duna ka bay mga records bahin sa panglawas sa namatay ?

IF NO, REFUSES TO ANSWER OR DON'T KNOW GO TO 6.9.

1	Yes	|___|
2	No	|___|
8	Refuses to answer	|___|
9	Don't know	|___|

6.5. Mahimo ba nakong makita kining mga records?
IF NO, REFUSES TO ANSWER GO TO 6.10. IF YES AND RESPONDENT ALLOWS YOU TO SEE THE RECORDS, TRANSCRIBE THE RECORDS.

1	Yes	|___|
2	No	|___|
8	Refuses to answer	|___|
9	Don't know	|___|

6.6. RECORD THE DATE OF THE TWOMOST RECENT VISITS.

1 |___|___|/|___|___|/|___|___|___|___|
Mm dd yyyy

2 |___|___|/|___|___|/|___|___|___|___|
Mm dd yyyy

IF NOT LISTED, MARK 999

6.7. RECORD THE DATE OF THE LAST NOTE

|___|___|/|___|___|/|___|___|___|___|
Mm dd yyyy

6.8. TRANSCRIBE THE NOTE

6.9.	Gi-isyuhan ka ba ug death certificate ? **IF NO, REFUSES TO ANSWER OR DON'T KNOW, GO TO SECTION 7.**	1<br>2<br>8<br>9	Yes<br>No<br>Refuses to answer<br>Don't know	|___| |___| |___| |___|
6.10.	Mahimo bang makita nako ang death certificate? **IF NO, GO TO SECTION 7.**	1<br>2<br>8	Yes<br>No<br>Refuses to answer	|___| |___| |___|
6.11.	Record the immediate cause	______________________<br>______________________<br>______________________		|___|
6.12.	Record the first underlying cause of death from the certificate.	______________________<br>______________________<br>______________________		|___|
6.13.	Record the 2nd underlying cause of death from the certificate.	______________________<br>______________________		|___|
6.14.	Record the 3rd underlying cause of death from the certificate.	______________________<br>______________________<br>______________________<br>______________________		|___|
6.15	Record the contributing cause of death from the certificate.	______________________<br>______________________<br>]______________________		|___|

SECTION 6. OPEN ENDED RESPONSE AND INTERVIEWERS COMMENTS AND OBSERVATIONS

INTRUCTIONS TO INTERVIEWERS: Ask the respondent: Thank you for the patient responses to this exhaustive set of questions. Could you please summarize or tell us in your own words, any additional information about the illness and/or death of your loved one?

TO THE INTERVIEWER: Write down what the respondent tells you in his/her own words. Do not prompt except for asking whether there was anything else after the respondent finishes. While recording, underline any unfamiliar terms. You may also use this space to write down your comments and observations about the interview.

END OF INTERVIEW
THANK THE RESPONDENT FOR PARTICIPATION.

SECTION 6. OPEN ENDED RESPONSE AND INTERVIEWERS COMMENTS AND OBSERVATIONS

INTRUCTIONS TO INTERVIEWERS: Translate what you have written in the previous page into English.

